# Identification of 6-amino-1*H*-pyrazolo[3,4-*d*]pyrimidines with *in vivo* efficacy against visceral leishmaniasis†
†Electronic supplementary information (ESI) available. See DOI: 10.1039/d0md00203h


**DOI:** 10.1039/d0md00203h

**Published:** 2020-08-06

**Authors:** Michael G. Thomas, Manu De Rycker, Myriam Ajakane, Sabrinia D. Crouch, Lorna Campbell, Alain Daugan, Gloria Fra, César Guerrero, Claire J. Mackenzie, Lorna MacLean, Sujatha Manthri, Franck Martin, Suzanne Norval, Maria Osuna-Cabello, Jennifer Riley, Yoko Shishikura, Juan Miguel-Siles, Frederick R. C. Simeons, Laste Stojanovski, John Thomas, Stephen Thompson, Raul F. Velasco, Jose M. Fiandor, Paul G. Wyatt, Kevin D. Read, Ian H. Gilbert, Timothy J. Miles

**Affiliations:** a Drug Discovery Unit , Wellcome Centre for Anti-Infectives Research , Division of Biological Chemistry and Drug Discovery , School of Life Sciences , University of Dundee , Dundee DD1 5EH , UK . Email: I.H.Gilbert@dundee.ac.uk; b Centre de Recherche , GlaxoSmithKline , Les Ulis, 25,27 Avenue du Quebec , 91140 Villebon sur Yvette , France; c Global Health R&D , GlaxoSmithKline , Calle Severo Ochoa, 2, 28760 Tres Cantos , Madrid , Spain . Email: tim.j.miles@gsk.com; d GalChimia S.A. , Cebreiro s/n, 15823, O Pino , A Coruña , Spain

## Abstract

Visceral leishmaniasis (VL) affects millions of people across the world, largely in developing nations. It is fatal if left untreated and the current treatments are inadequate. As such, there is an urgent need for new, improved medicines. In this paper, we describe the identification of a 6-amino-*N*-(piperidin-4-yl)-1*H*-pyrazolo[3,4-*d*]pyrimidine scaffold and its optimization to give compounds which showed efficacy when orally dosed in a mouse model of VL.

## Abstract 2

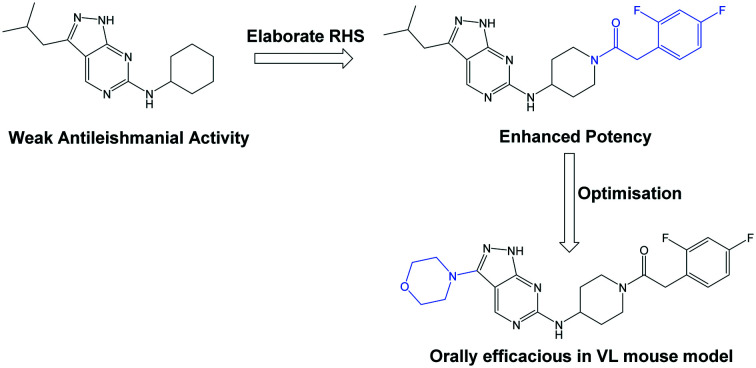
The development of a chemical series with oral efficacy against visceral leishmaniasis is described.

## Introduction

Visceral leishmaniasis (VL) is caused by infection with the protozoan parasites *L. donovani* and *L. infantum* and is typically fatal unless treated, infecting around 50 000–90 000 people annually and resulting in a death toll of between 20 000–30 000.^1^,^2^ Current therapies suffer from numerous issues such as high cost, problematic modes of dosing, and toxicity.^3^ In addition, the global development pipeline for VL is sparse with four additional new chemical entities (NCEs) just entering the early pre-clinical phase.^4^–^7^ There is therefore an urgent need for new therapeutic classes to help tackle this neglected disease.

**Fig. 1:**
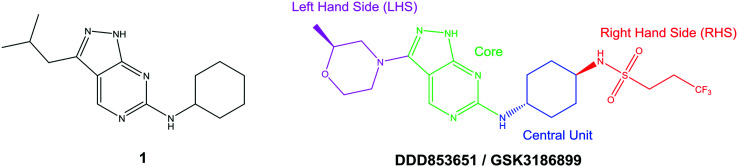
Initial active compound **1** and preclinical candidate **DDD853651**/**GSK3186899**.

The primary objective of this program was therefore to identify safe, effective, oral, short-course (ideally ≤10 days) drug candidates for VL, in line with the DND*i* (Drugs for Neglected Disease *initiative*) target product profile.^8^ Due to a lack of validated druggable targets for VL, we focused on chemical series that showed antiparasitic activity in an assay involving parasites growing in mammalian cells, aiming to progress these into *in vivo* efficacy studies, where they could be bench-marked against miltefosine; this currently being the only available oral therapy for VL. This phenotypic approach was used successfully for the identification of two preclinical candidates.^5^,^7^ One of these, **DDD853651**/**GSK3186899** was developed from pyrazolopyrimidine **1**, with a key step being elaboration of the cyclohexylamine to a sulfonamide substituted 1,4-*trans*-cyclohexyldiamine (Fig. 1). In this work, we describe further modifications to the cyclohexylamine, leading to a series of substituted 4-aminopiperidines which displayed efficacy in a mouse model of VL.

## Results and discussion

For the identification of **DDD853651**/**GSK3186899**, which contained a *trans*-1,4-cyclohexyldiamine, a key strategy was to optimise the balance between potency in the intracellular *Leishmania* assay (*L. donovani* in THP-1 cells, referred to as Ld InMac assay^9^) and solubility.^10^ In that case, we focused on **2** (Table 1), where the sulfonamide was required for potency but was poorly soluble. We noted that amide **3**, whilst inactive, did show improved aqueous solubility (51 *vs.* 1 μM), so we investigated alternative central units to identify start points which retained activity and showed improved solubility compared to **2**. To this end, we examined a 4-aminopiperidine central unit with both sulfonamide and amide substituents (Table 1). In contrast to the *trans*-1,4-cyclohexyldiamine series, the equivalent sulfonamide was >10-fold less active (**5***cf.***2**), whilst amide **4** was >10-fold more active than its comparator (**4***cf.***3**) and showed similar aqueous solubility (59 μM *cf.* 51 μM). We therefore selected **4** as a suitable start-point for further chemistry. As previously discussed,^10^ in order to progress to *in vivo* studies we targeted compounds with pEC_50_ > 5.8, solubility > 100 μM and intrinsic clearance (Cl_i_) < 5.0 ml min^–1^ g^–1^ (mouse liver microsomes), so we explored the SAR around **4**, initially focusing on the amide substituent. We noted that either lengthening the linker to the aromatic ring, or removing it, led to a loss of activity (**6** and **7** respectively). Substitution of the phenylacetamide led to 2,4-difluorinated analogue **8** with an increase in potency compared to **4**, whilst other changes, such as the dichloro analogue **9** and α-methyl analogue **10** failed to improve potency, although **10** did show improved aqueous solubility, possibly due to the introduction of a sp^3^ centre.

**Table 1:** SAR of analogues with iso-butyl LHS

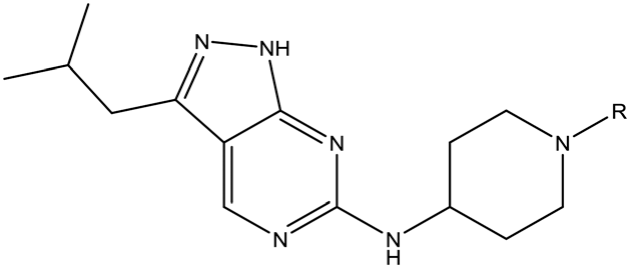					
R	Compound	Ld InMac^*a*^ pEC_50_	THP-1^*a*^ pEC_50_	Aqueous solubility^*b*^ μM	Cl_i_ mouse^*c*^ ml min^–1^ g^–1^
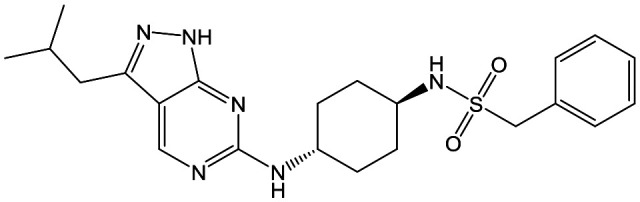	**2**	6.2	<4.3	<1	27
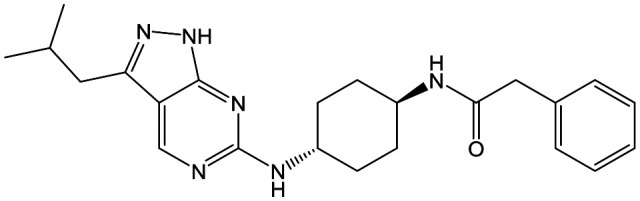	**3**	<4.3	<4.3	51	ND^*d*^
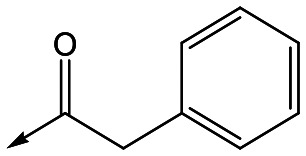	**4**	5.1	<4.3	59	18
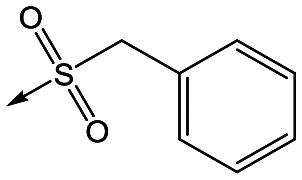	**5**	4.9	<4.3	10	>50
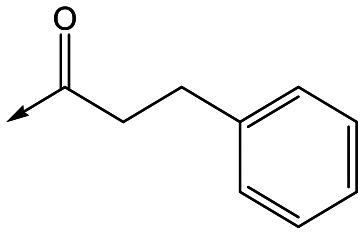	**6**	4.7	<4.3	ND^*d*^	ND^*d*^
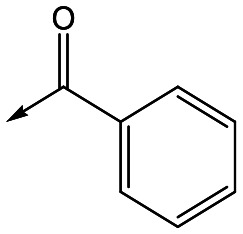	**7**	<4.3	<4.3	291	7
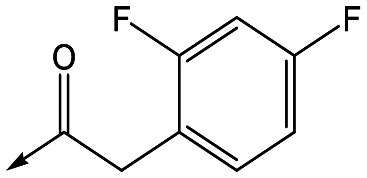	**8**	5.8	<4.3	26	12
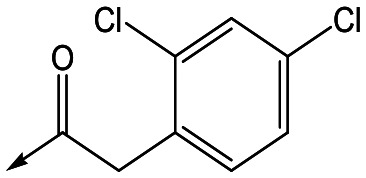	**9**	5.0	<4.3	26	30
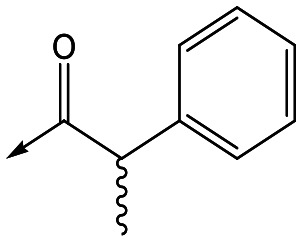	**10** **(rac)**	5.3	<4.3	140	ND^*d*^

^*a*^Ld InMac is the intramacrophage assay carried out in THP-1 cells with *L. donovani* amastigotes.^9^ Data are the mean values for *n* ≥ 3 replicates and standard deviations are ≤0.3.

^*b*^Aq. solubility is kinetic aqueous solubility (CAD).^11^

^*c*^Cl_i_ is mouse liver microsomal intrinsic clearance.^10^

^*d*^ND means not determined.

With 2,4-difluorophenylacetamide **8** as the most potent RHS (right hand side, see Fig. 1 in the introduction) identified, we explored the SAR (structure activity relationship) around the LHS (left hand side, see Fig. 1 in the Introduction) to improve solubility and metabolic stability, initially focusing on alkyl substituents (Table 2). Neopentyl **11** was inactive whilst the smaller cyclopropyl group of **12** maintained potency and displayed improved metabolic stability. Changing the cyclopropyl group for tetrahydropyran (THP) led to **13**, with a pEC_50_ value of 6.0, a small improvement in solubility, and excellent metabolic stability. To further improve the physicochemical characteristics of the series, we examined the methylene-linked morpholine **14** which also showed excellent metabolic stability, although it was only weakly active. Removing the methylene linker gave the directly attached morpholine **15**, which retained *in vitro* potency and metabolic stability, whilst switching to O-linked compound **16** gave a drop in activity.

**Table 2:** SAR of analogues with *N*-(2,4-difluorophenylacetyl)piperidine RHS

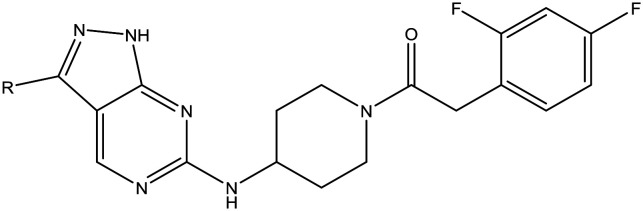					
R	Compound	Ld InMac pEC_50_^*a*^	THP-1^*a*^ pEC_50_	Aqueous solubility^*b*^ μM	Cl_i_ mouse,^*c*^ ml min^–1^ g^–1^
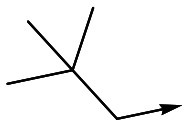	**11**	<4.3	<4.3	27	22.0
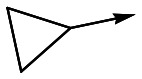	**12**	5.9	<4.3	39	5.0
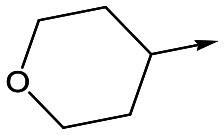	**13**	6.0	<4.3	78	0.5
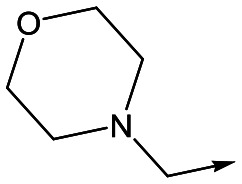	**14**	4.6	<4.3	ND^*d*^	<0.5
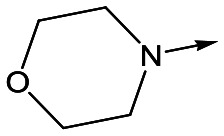	**15**	6.1	<4.3	14	2
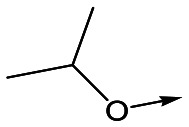	**16**	5.2	<4.3	33	ND^*d*^
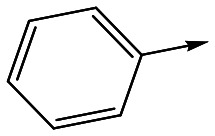	**17**	6.5	<4.3	<1	7.2
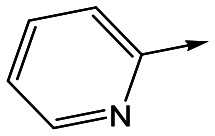	**18**	6.1	<4.3	23	6.7
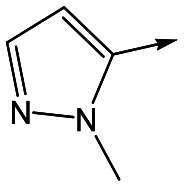	**19**	5.5	4.7	30	3.3
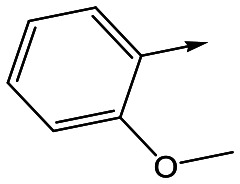	**20**	7.1	4.4	<1	5.0
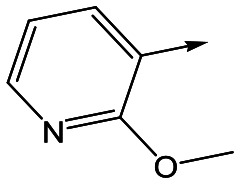	**21**	7.1	<4.3	25	15

^*a*^Ld InMac is the intramacrophage assay carried out in THP-1 cells with *L. donovani* amastigotes.^9^ Data are the mean values for *n* ≥ 3 replicates and standard deviations are ≤0.4.

^*b*^Aq. solubility is kinetic aqueous solubility (CAD).^11^

^*c*^Cl_i_ is mouse liver microsomal intrinsic clearance.^10^

^*d*^ND means not determined.

To further explore the SAR around **13**, with a cyclic substituent on the LHS, a series of aromatic groups were explored. Phenyl substituted **17** showed an increase in potency compared to **9**, although it was hampered by poor solubility; this was improved slightly by moving to the more polar pyridyl analogue **18**, and moving to 5-membered heteroaromatics such as **19** also improved metabolic stability, albeit with a 10-fold loss in potency. A variety of other 5 and 6-membered heteroaromatics were explored, but none led to significant improvements (data not shown). Substitution on the aromatic ring of **17** was also investigated, leading to the 2-methoxyphenyl analogue **20**, with a pEC_50_ value of 7.1 in the Ld InMac assay. This was one of the most potent analogues in the series, comparing very favourably with the standard treatments for VL, amphotericin B and miltefosine (pEC_50_'s of 6.7 and 6.1 respectively). In an attempt to further improve solubility, methoxypyridyl analogue **21** was synthesized; whilst potency of **20** was maintained and solubility was improved, poorer microsomal stability was observed.

**Fig. 2:**
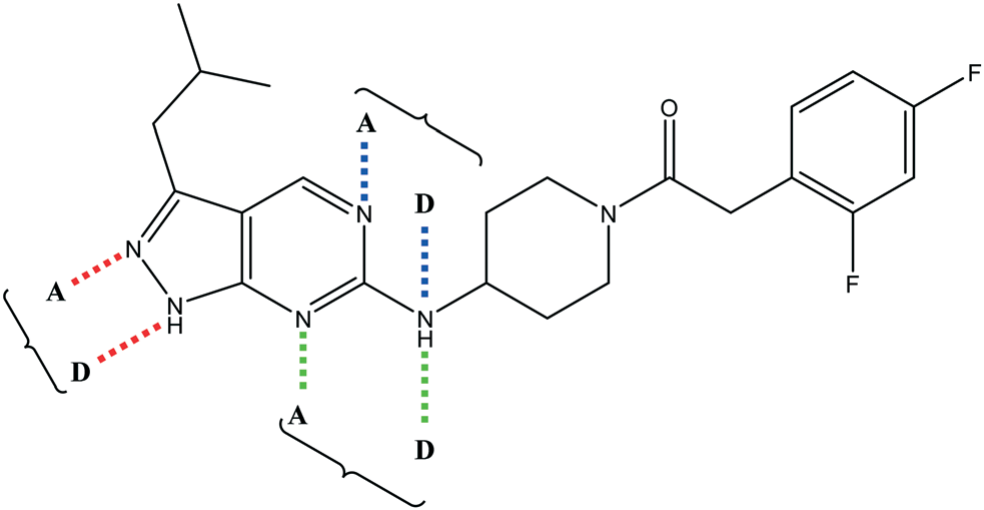
Hydrogen bond donor–acceptor pairs in **8**.

Having demonstrated that variations to the RHS (Table 1) and LHS (Table 2) could deliver compounds with a good balance of potency, solubility and metabolic stability, we switched our attention to the core pyrazolopyrimidine and the central unit (Table 3). Although we later elucidated the target of the series as being Leishmania-CRK12 (cyclin related kinase 12),^5^ at this point we were reliant on phenotypic drug discovery strategies to drive compound design. We were aware that intermolecular hydrogen bonding between the donor–acceptor pairs within the core was likely to be detrimental to solubility (Fig. 2), as was the planarity of the compounds.^10^ We therefore synthesized a set of analogues to probe the SAR around the various hydrogen bond donor–acceptor pairs in the core, and also examined a further series of central units to identify any that could maintain potency whilst adding more flexibility and three-dimensional character.

**Table 3:** SAR around the core and central unit

	Compound	Comparator	Ld InMac pEC_50_^*a*^	THP-1 pEC_50_^*a*^
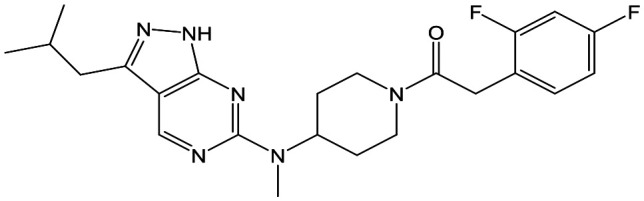	**22**	**9**	<4.3	<4.3
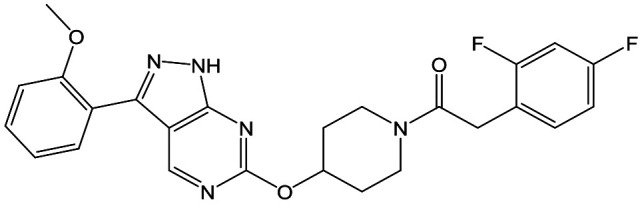	**23**	**20**	4.7	<4.3
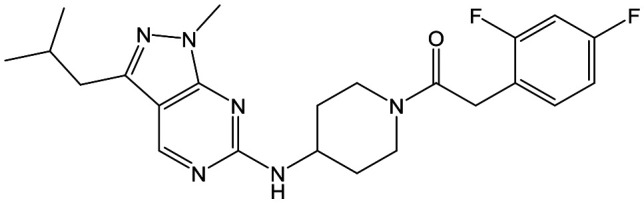	**24**	**9**	<4.3	<4.3
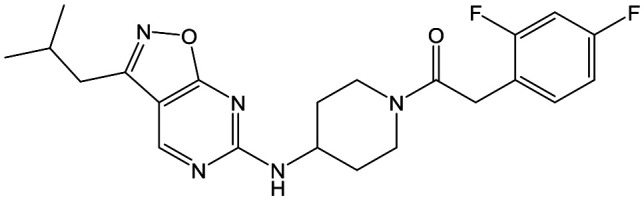	**25**	**9**	<4.3	<4.3
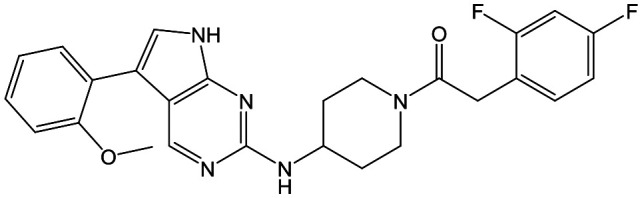	**26**	**20**	<4.3	<4.3
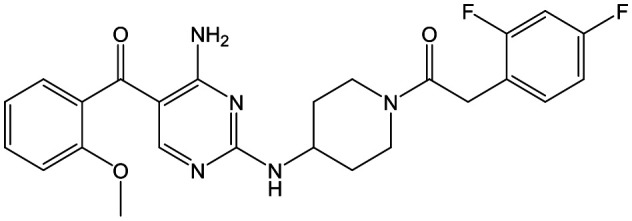	**27**	**20**	5.2	5.0
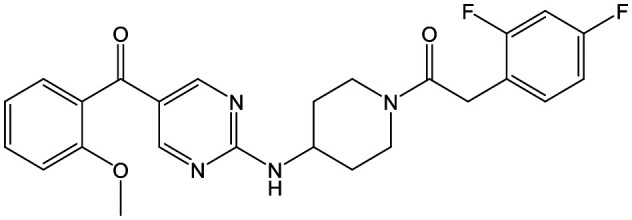	**28**	**20**	5.2	<4.3
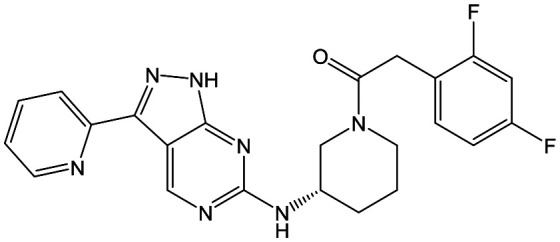	**29** **(*S*)**	**18**	4.9	<4.3
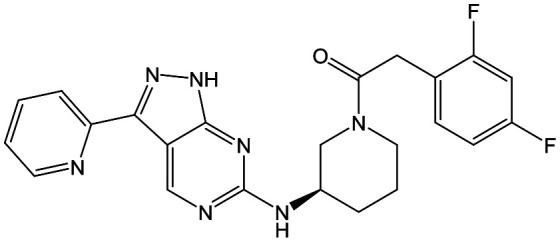	**30** **(*R*)**	**18**	<4.3	<4.3
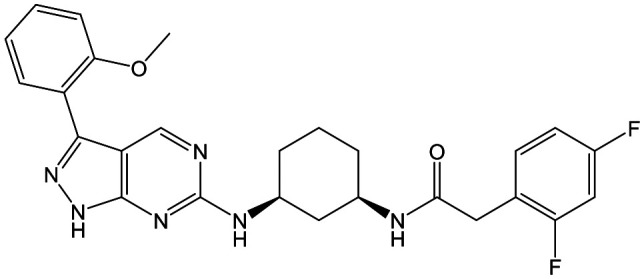	**31** **(*cis*)**	**20**	4.5	4.6
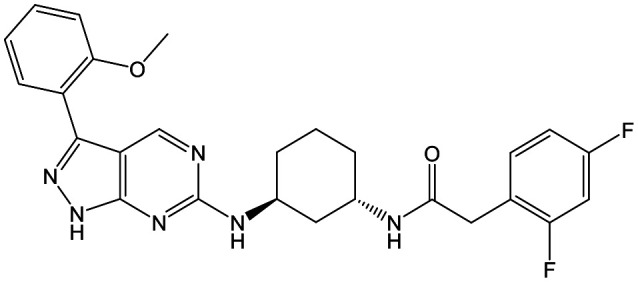	**32** **(*trans*)**	**20**	4.8	4.5
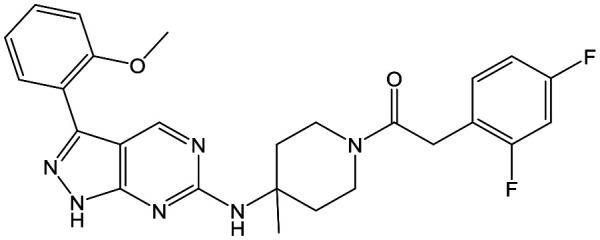	**33**	**20**	5.4	4.4
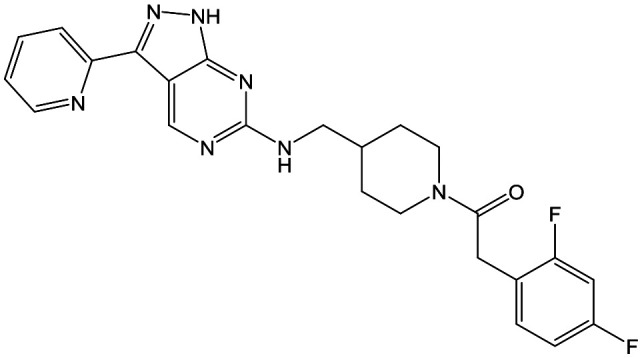	**34**	**18**	<4.3	<4.3
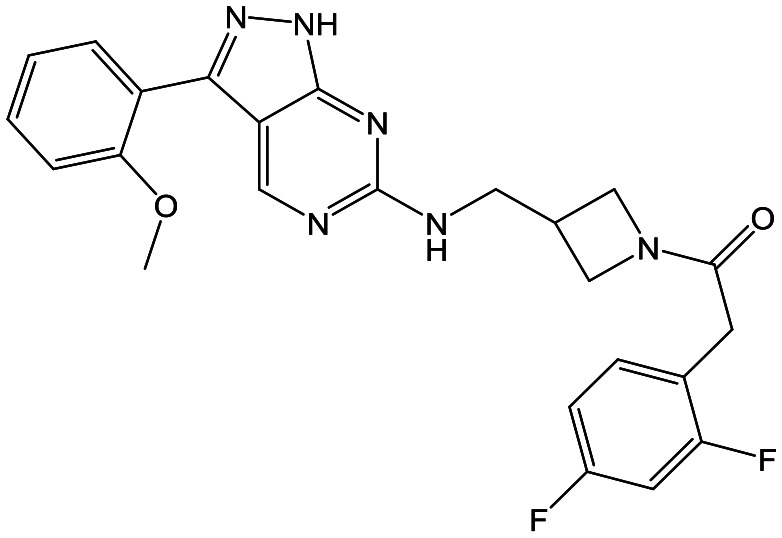	**35**	**20**	<4.3	<4.3
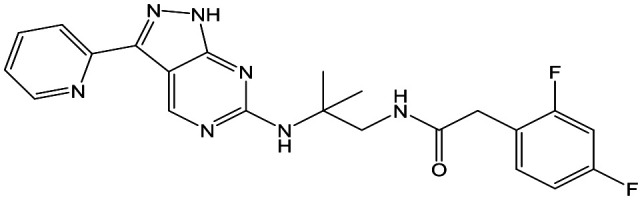	**36**	**18**	<4.3	<4.3

^*a*^Ld InMac is the intramacrophage assay carried out in THP-1 cells with *L. donovani* amastigotes.^9^ Data are the mean values for *n* ≥ 3 replicates and standard deviations are ≤0.3.

Alkylation of the aminopyrimidine N–H or replacement by oxygen to give **22** and **23** respectively, led to a >10-fold reduction in potency compared to its' matched molecular pair (compounds **9**, **18** and **20** were all used as comparators for this exercise). Additionally, alkylation of the pyrazole N–H (**24**) as well as replacement of the pyrazole with isoxazole (**25**) or pyrrole (**26**) all led to a >10-fold loss of potency. The pyrazole ring was opened to give pyridimines **27** and **28**, leading to decreased potency and also toxicity against the host THP-1 cells in the case of **27**.

Switching attention to the central unit, 3-aminopiperidine enantiomers **29** and **30** and the 1,3-diaminocyclohexyl analogues **31** and **32** were either weakly active or inactive, as was methylated aminopiperidine **33**. More flexible linkers such as aminomethylpiperidine **34**, aminomethylcyclobutyl **35** and ethylenediamine **36** were also inactive. Further diverse linkers, with numerous substituents (amides and sulfonamides) were tested, but all proved to be inactive beyond the already identified piperidines and 1,4-*trans*-cyclohexylamines (data not shown).

Refocusing on **20**, where the introduction of the 2-methoxyphenyl LHS gave a significant improvement in potency, further RHS piperidine substituents were explored (Table 4). Introduction of the trifluoropropylsulfonamide from the preclinical candidate (**DDD853651**/**GSK3186899**) did give a potent compound (**37**) although with very poor solubility and intrinsic clearance; this was similar for carbamate linked compounds **38** and **39**. Conversely, urea linked compounds such as **40** maintained reasonable solubility and metabolic stability but reduced potency. Also, directly attaching a heterocycle led to **41** with very good potency but low solubility. Finally we examined a further set of amides, identifying cyclopropylmethyl analogue **42** with good potency and metabolic stability, and 3,4-difluorophenyl analogue **43** with improved potency and solubility compared to **20**.

**Table 4:** SAR of analogues with 2-methoxyphenyl LHS

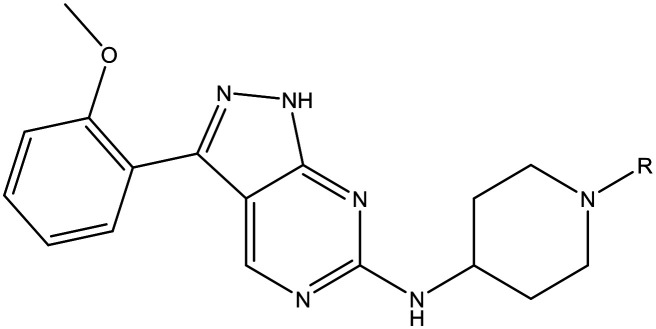					
R	Compound	Ld InMac pEC_50_^*a*^	THP-1 pEC_50_^*a*^	Aqueous solubility^*b*^ μM	Cl_i_ mouse,^*c*^ ml min^–1^ g^–1^
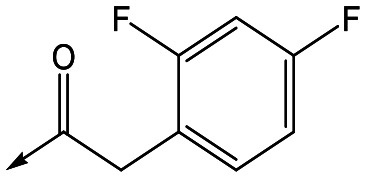	**20**	7.1	4.4	<1	5.0
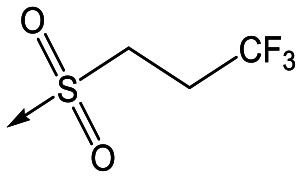	**37**	6.7	<4.3	<1	14
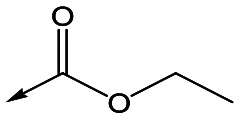	**38**	6.3	4.4	24	14
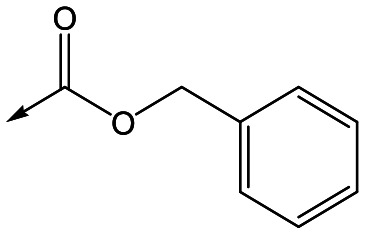	**39**	6.4	<4.3	ND^*d*^	9
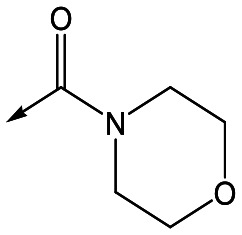	**40**	5.7	<4.3	79	2
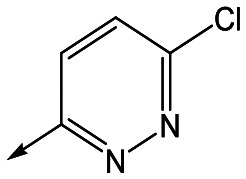	**41**	6.8	<4.3	4^12^	3.4
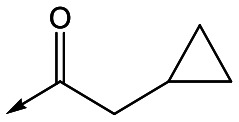	**42**	6.5	4.4	22	1.6
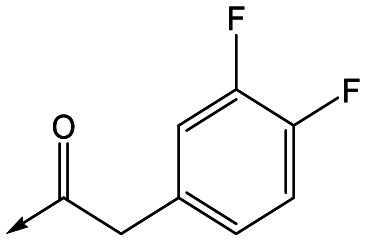	**43**	7.6	4.8	53	6.3

^*a*^Ld InMac is the intramacrophage assay carried out in THP-1 cells with *L. donovani* amastigotes.^9^ Data are the mean values for *n* ≥ 3 replicates and standard deviations are ≤0.4.

^*b*^Aq. solubility is kinetic aqueous solubility (CAD).^11^

^*c*^Cl_i_ is mouse liver microsomal intrinsic clearance.^10^

^*d*^ND means not determined.

Utilising the SAR understanding we had developed, a set of analogues were synthesized that combined the most interesting LHS and RHS (Table 5). Maintaining the cyclopropylmethyl amide of **42** led to the THP (**44**), 2-pyridyl (**45**) and morpholine (**46**) analogues that were only moderately potent but were much more soluble, with **44** also having very good metabolic stability. Maintaining the 3,4-difluorophenyl RHS led to THP **47** and morpholine **48**, both of which had reasonable potency, high solubility and good metabolic stability. Finally, the chloropyridazine RHS led to THP analogue **49** which was reasonably potent and morpholine **50** which again showed good potency, solubility and metabolic stability.

**Table 5:** Designed analogues based on key SAR

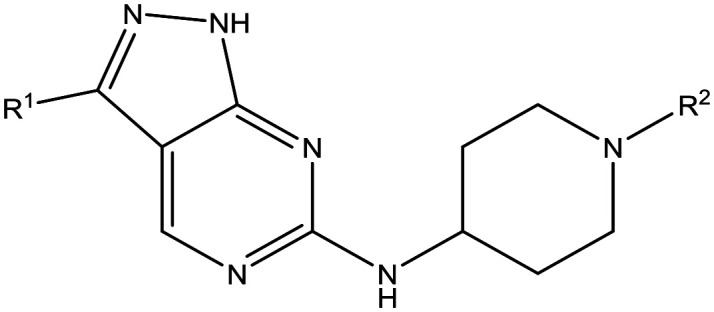						
R^1^	R^2^	Compound	Ld InMac pEC_50_^*a*^	THP-1 pEC_50_^*a*^	Aqueous solubility^*b*^ μM	Cl_i_ mouse^*c*^ ml min^–1^ g^–1^
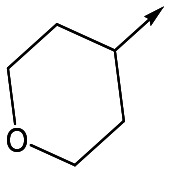	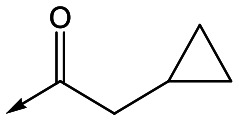	**44**	5.1	<4.3	≥496	0.8
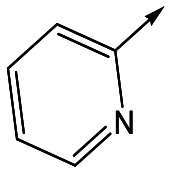	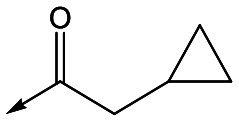	**45**	5.3	<4.3	≥392	ND^*d*^
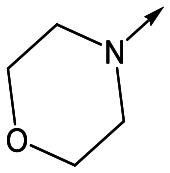	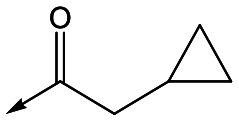	**46**	4.8	<4.3	ND^*d*^	ND^*d*^
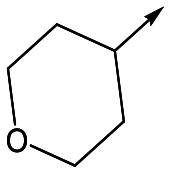	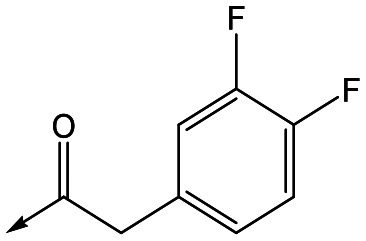	**47**	6.0	<4.3	≥429	3.1
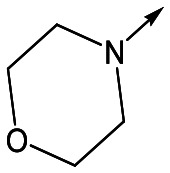	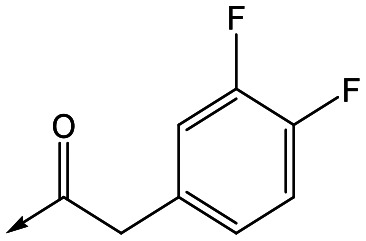	**48**	6.1	<4.3	≥465	1.6
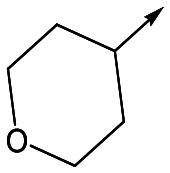	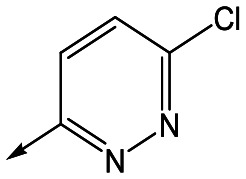	**49**	5.8	<4.3	63^12^	ND^*d*^
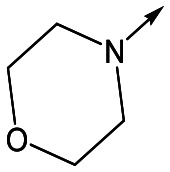	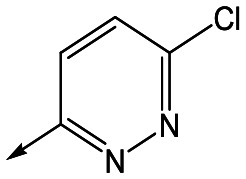	**50**	6.2	<4.3	121^12^	0.6

^*a*^Ld InMac is the intramacrophage assay carried out in THP-1 cells with *L. donovani* amastigotes.^9^ Data are the mean values for *n* ≥ 3 replicates and standard deviations are ≤0.4.

^*b*^Aq. solubility is kinetic aqueous solubility (CAD).^11^

^*c*^Cl_i_ is mouse liver microsomal intrinsic clearance.^10^

^*d*^ND means not determined.

To select the most suitable compounds to progress to *in vivo* studies, the fasted state simulated intestinal fluid (FaSSIF) solubility of **20**, **42**, **48** and **50** was measured,^13^ as this had proved to be successful for triaging compounds in the 1,4-*trans*-cyclohexyldiamine series.^10^**20**, the most potent compound with Cl_i_ ≤ 5.0 ml min^–1^ g^–1^, and **42**, which had moderate aqueous solubility, both had FaSSIF solubility of <1 μg ml^–1^, whereas **48** and **50** had FaSSIF solubilities of 97 and 88 μg ml^–1^ respectively. On this basis, **48** and **50** were progressed into orally dosed pharmacokinetic studies in female Balb-c mice (Table 6), with both having sufficient *C*_max_ (the highest concentration of a drug in the blood) and AUC (area under the curve) to progress into the previously reported mouse efficacy model of VL.^10^

**Table 6:** Pharmacokinetic profile of compounds **48** and **50** in Balb-c mice, dosed orally

Compound	Dose mg kg^–1^	AUC ng min ml^–1^	*C*_max_ ng ml^–1^	*T*_max_ h
**48**	100	1 253 767	5765	1
**50**	100	2 388 964	5229	4

Both compounds were dosed orally for 5 days (Table 7), with **48** giving 60% reduction of parasite liver load at the lower dose, and 98% reduction at the higher dose, and **50** giving 17% reduction at the lower dose and 57% at the higher dose. For **48**, this met our criteria for progression towards candidate selection and it was therefore profiled more fully.

**Table 7:** Mouse efficacy model of VL for **48** and **50**

Compound	Dosing regimen	% Suppression of parasite load
**48**	30 mg kg^–1^*b.i.d.* for 5 days PO	60
**48**	100 mg kg^–1^*b.i.d.* for 5 days PO	98
**50**	25 mg kg^–1^*b.i.d.* for 5 days PO	17
**50**	50 mg kg^–1^*b.i.d.* for 5 days PO	57

At this point, the target of the series was demonstrated to be the parasitic kinase Leishmania-CRK12, so to fully understand kinase selectivity **48** was further profiled against a panel of 140 human kinase receptors at 10 μM concentration.^14^ From this, only 4 kinases were inhibited at >40% (ERK8 [extracellular signal-regulated kinases], p38α and p38β MAPK [mitogen-activated protein kinases] and CDK2 [cyclin dependant kinase]). Full IC_50_ curves were generated for these, showing that the kinases most affected were ERK8 (IC_50_ = 0.11 μM) and p38β MAPK (IC_50_ = 0.55 μM). We didn't consider these results to preclude further development of **48**.

A further *in vivo* study demonstrated that **48** had a mean liver : blood ratio of 4.8 : 1 which could prove beneficial, as the liver is one of the major sites of parasitic burden in VL infections.^2^**48** was also progressed to a rat dose-escalation study in order to determine whether exposures higher than the efficacious exposure could be achieved (Table 8); this would be critical in order to progress the compound to rat toxicology studies. In this case, increasing the dose from 100 to 300 mg kg^–1^ led to only a 1.2-fold increase in *C*_max_ and a 1.7-fold increase in AUC_(0-Tlast)_ suggesting that it would be challenging to determine a therapeutic index in future toxicological studies.

**Table 8:** Rat PK data for **48** at 10, 100 and 300 mg kg^–1^

Dose (mg kg^–1^)	*C*_max_ (ng ml^–1^)	*T*_max_ (h)	AUC _(0-Tlast)_ (ng h ml^–1^)
10	229	0.67	488
100	5520	6.00	50621
300	6760	6.67	84647

**Scheme 1:**
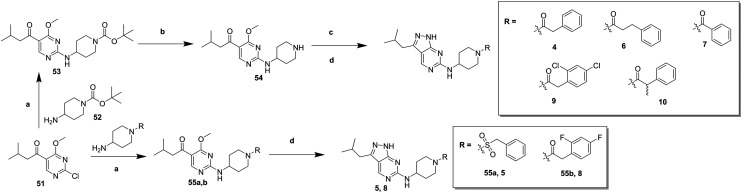
Synthesis of compounds **4–10**. (a) *N*,*N*-Diisopropylethylamine (DIPEA), butan-1-ol; (b) trifluoroacetic acid (TFA), dichloromethane (DCM), 97% (over 2 steps); (c) R–COOH, *N*,*N*,*N*′,*N*′-tetramethyl-*O*-(benzotriazol-1-yl)uronium tetrafluoroborate (TBTU), DIPEA, DCM; (d) hydrazine hydrate, butan-1-ol, 30–56% (over 2 steps).

**Scheme 2:**
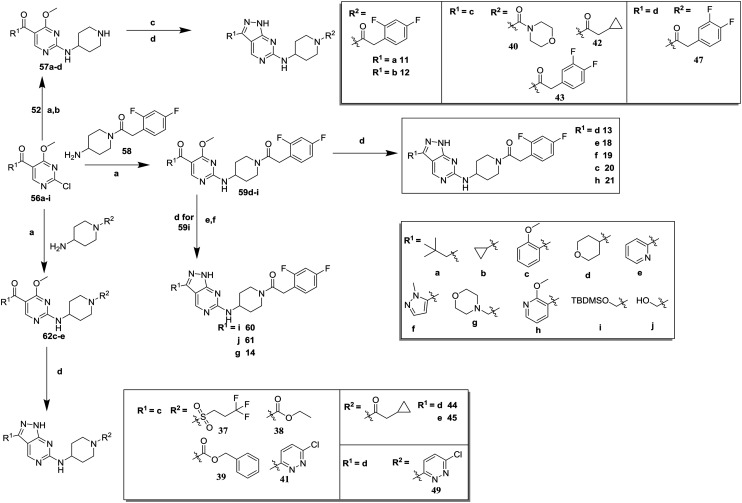
Synthesis of compounds **11–21**, **37–45**, **47** and **49**. (a) DIPEA, butan-1-ol; (b) TFA, DCM, 47–83% (over 2 steps); (c) R^2^–COOH, TBTU, DIPEA, dimethylformamide (DMF); (d) hydrazine hydrate, butan-1-ol, 22–48% (over 2 steps); (e) tetra-*n*-butylammonium fluoride (TBAF), THF, 86%; (f) methanesulfonyl chloride (MsCl), DIPEA, acetonitrile (MeCN) then morpholine, 18%.

**Scheme 3:**
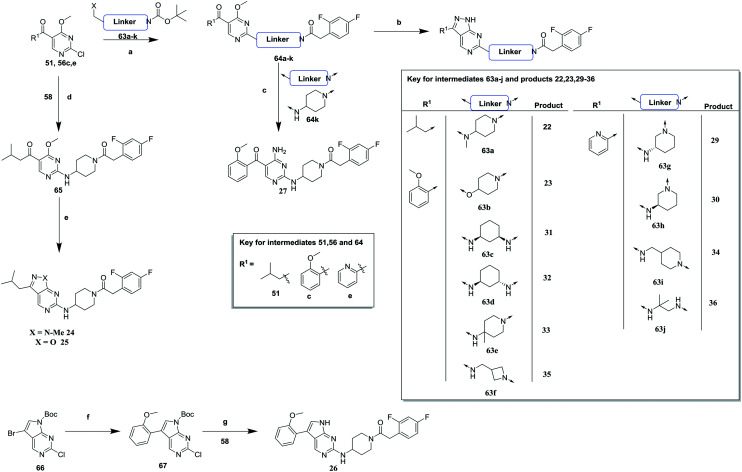
Synthesis of compounds **22**, **23**, **26**, **29–36**. (a) (i) DIPEA, butan-1-ol (ii) TFA, DCM (iii) TBTU, DIPEA, DCM; (b) hydrazine hydrate, butan-1-ol, 14–43% (over steps a and b); (c) ammonium hydroxide, ethanol (EtOH), 83%; (d) DIPEA, butan-1-ol; (e) (i) methylhydrazine, DIPEA, butan-1-ol (for **24**), 18% (over 2 steps) (ii) hydroxylamine. Hydrogen chloride (HCl), DIPEA, butan-1-ol (for **25**), 10% (over 2 steps); (f) (i) (2-methoxyphenyl)boronic acid, potassium phosphate (K_3_PO_4_), bis(di-*tert*-butyl(4-dimethylaminophenyl)phosphine)dichloropalladium(ii), MeCN (ii) Boc_2_O, sodium hydride (NaH), THF, 92%; (g) DIPEA, *N*-methyl-2-pyrrolidone (NMP), 17%.

**Scheme 4:**
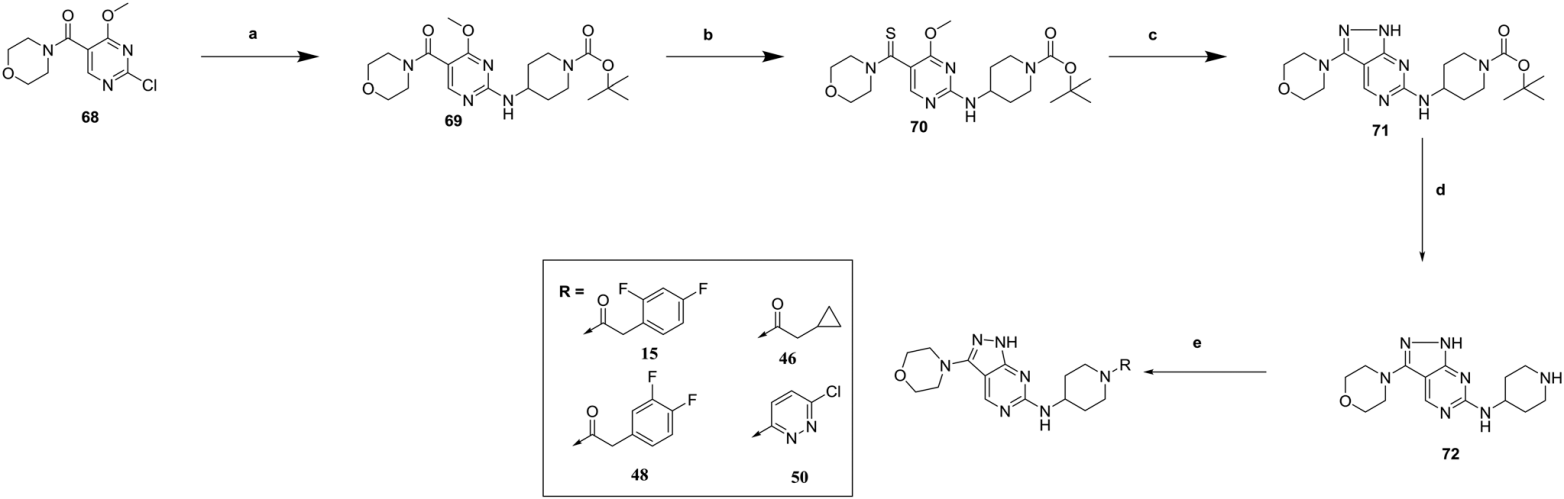
Synthesis of **15**, **46**, **48** and **50**. (a) 1-Boc-4-aminopiperidine, DIPEA, butan-1-ol, 67%; (b) Lawessons reagent, THF, 86%; (c) hydrazine hydrate, 1,4-dioxane, 94% (d) TFA/DCM, 100% (e) (i) R–COOH, TBTU, DIPEA, DMF (for **15**, **46** and **48**) (ii) 3,6-dichloropyridazine, DIPEA, EtOH (for **50**), 36–43%.

In order to synthesise the compounds described, a number of different synthetic routes were utilized, as outlined in Schemes 1–4. Scheme 1 shows the synthetic route that was used to access compounds **4–10**, where the readily accessible 2-chloro-4-methoxy pyrimidine **51**^10^ was treated with the Boc (*tert*-butyloxycarbonyl protecting group) protected aminopyrimidine **52** to give **53**, which was deprotected under acidic conditions to give **54**. This was subsequently coupled with a relevant acid then cyclized with hydrazine to yield analogues **4–7** and **9–10**. Alternately, an elaborated aminopiperidine could be coupled with **51** to give **55a** (R = benzylsulfonyl) or **55b** (R = 2,4-difluorobenzoyl) which were cyclized to give compounds **5** and **8** respectively.

Compounds **11–21**, with the 2,4-difluorophenacetamide RHS and variations to the LHS were synthesized according to Scheme 2, such that **56**, with an appropriate R-group (R^1^ = **a–d** in table), was coupled to **52** and the resulting intermediate Boc-deprotected by treatment with TFA to give **57a–d**. **57a** and **57b** were coupled to 2,4-difluorophenylacetic acid and subsequently cyclized with hydrazine to give **11** and **12**. Compounds **40**, **42**, **43** and **47** were synthesized by a similar procedure using **57c** or **d** and a suitable acid. Alternately, **56d–g** were coupled directly with the elaborated aminopiperidine **58** to give **59d–g** and cyclized with hydrazine to give compounds **13** and **18–21**. **14** was synthesized by a similar route, but after **59i** was cyclised to give **60**, deprotection of the intermediate TBDMS alcohol gave **61** which was mesylated and displaced with morpholine. Finally, by choosing suitably substituted aminopiperidines alongside the relevant **56**, intermediates **62c–e** were generated and cyclized to give compounds **37–39**, **41**, **44**, **45** and **49**.

Scheme 3 highlights the synthetic routes to compounds with variations to the linking group between the pyrimidine core and the RHS amide (**22–36**). For compounds **22**, **23** and **29–36**, a suitably substituted 2-chloro-4-methoxy pyrimidine (**51** or **56c**, **e**) was treated with the relevant mono-Boc protected diamine **63a–j**. These were subsequently deprotected and coupled with 2,4-difluorophenylacetic acid to give **64a–j** which were cyclized with hydrazine to give the relevant compound. Alternatively, treatment of **64k** with ammonia gave the monocyclic analogue **27**. For compounds **24** and **25**, **65** was generated from **56a** and **58** then cyclized with either methylhydrazine or hydroxylamine to give **24** and **25** respectively. Replacement of the pyrazolopyrimidine scaffold with a 7*H*-pyrrolo[2,3-*d*]pyrimidine required a separate synthesis, whereby Boc protected **66** was cross-coupled with 2-methoxyphenylboronic acid and subsequently Boc-reprotected to give **67** which was coupled with **58** and then Boc deprotected to give **26**.

Finally, the 3-morpholinopyrazole analogues **15**, **46**, **48** and **50** were synthesized *via* cyclisation of relevant thioamides as previously described,^10^ and shown in Scheme 4. **68** was therefore treated with **52** to give **69**. Treatment with Lawessons reagent led to thioamide **70** which was cyclized with hydrazine to give **71**, with subsequent Boc deprotection yielding **72**. This was coupled with the relevant acid to give **15**, **46** and **48**, or treated with 3,6-dichloropyridazine to give **50**.

In summary, an aminopiperidine subseries of the previously disclosed pyrazolopyrimidines was identified as having potent *in vitro* antileishmanial activity. SAR exploration demonstrated that the core and central unit were optimal for activity, whilst optimization of the RHS and LHS led to **48**, with *in vivo* efficacy suitable for progression towards preclinical candidate selection. Whilst further profiling of **48** showed that it had a good safety profile, with only relatively weak inhibition of four human kinases highlighted, a dose escalation study in rats showed that **48** had infra-linear increase in exposure; this, alongside a minimum efficacious dose of 100 mg kg^–1^ meant that the compound was not progressed further. Despite this, the aminopiperidine subseries demonstrated the potential to deliver an alternative Leishmania CRK12 inhibitor for the treatment of VL.

## Ethical statements

### Mouse and rat pharmacokinetics

All animal studies were ethically reviewed and carried out in accordance with Animals (Scientific Procedures) Act 1986 and the GlaxoSmithKline (GSK)/Dundee University policies on the care, welfare, and treatment of animals.

### *In vivo* efficacy

All regulated procedures, at the University of Dundee, on living animals were carried out under the authority of a project license issued by the Home Office under the Animals (Scientific Procedures) Act 1986, as amended in 2012 (and in compliance with EU Directive EU/2010/63). License applications will have been approved by the University's Ethical Review Committee (ERC) before submission to the Home Office. The ERC has a general remit to develop and oversee policy on all aspects of the use of animals on University premises and is a subcommittee of the University Court, its highest governing body.

## Conflicts of interest

The following authors have shares in GlaxoSmithKline: M. A., S. D. C., A. D., F. M., J. M.-S., J. M. F., P. G. W., K. D. R., and T. J. M. The other authors declare no competing interests.

## Supplementary Material

Supplementary information
