# Supplementary material for: Identification of 6-amino-1H-pyrazolo[3,4-d]pyrimidines with in vivo efficacy against visceral leishmaniasis
Source: RSC Med Chem. 2020 Aug 6;11(10):1168–77. doi: 10.1039/d0md00203h (PMC7651859; doi:10.1039/d0md00203h)
Supplement: Supplementary file 1 [file MD-011-D0MD00203H-s001.pdf]

Electronic Supplementary Material (ESI) for RSC Medicinal Chemistry.
This journal is © The Royal Society of Chemistry 2020

Supplementary Information

Contents

Synthetic Procedures **S-1**

NMR and LCMS for Selected Compounds **S-28**

Assay Protocols **S-38**

Synthetic Procedures

General Experimental Information.

Chemicals and solvents were purchased from the Aldrich Chemical Company, Fluka, ABCR, VWR, Acros, Fluorochem and Alfa Aesar and were used as received. Air- and moisture-sensitive reactions were carried out under an inert atmosphere of argon in oven-dried glassware. Flash column chromatography was performed using pre-packed silica gel cartridges (230-400 mesh, 40–63 μm, from SiliCycle) using a Teledyne ISCO Combiflash Companion, or Combiflash Retrieve. ^{1}H NMR spectra were recorded on a Bruker Avance DPX 500 spectrometer. Chemical shifts (δ) are expressed in ppm recorded using the residual solvent as the internal reference in all cases. Signal splitting patterns are described as singlet (s), doublet (d), triplet (t), quartet (q), multiplet (m), broad (b), or a combination thereof. Coupling constants (*J*) are quoted to the nearest 0.1 Hz. LC-MS analyses were performed with either an Agilent HPLC 1100 series connected to a Bruker Daltonics MicrOTOF, or an Agilent Technologies 1200 series HPLC connected to an Agilent Technologies 6130 quadrupole LC/MS, where both instruments were connected to an Agilent diode array detector. All intermediates had a measured purity ≥ 90% and

all assay compounds had a measured purity of ≥ 95% as determined using this analytical LC-MS system (TIC and UV). High resolution electrospray measurements were performed on a Bruker Daltonics MicrOTOF mass spectrometer. Microwave-assisted chemistry was performed using a Biotage Initiator Microwave Synthesizer.

1-(4-((3-*iso*Butyl-1*H*-pyrazolo[3,4-*d*]pyrimidin-6-yl)amino)piperidin-1-yl)-2-phenylethan-1-one (4)

To **54** (50 mg, 0.17 mmol) in DCM (5 mL) was added DIPEA (66 μL, 0.34 mmol) and TBTU (66 mg, 0.21 mmol), stirred for 10 min and phenylacetic acid (26 mg, 0.19 mmol) added. After stirring for 18 h, RM was washed with water (5 mL), solvent evaporated and crude material dissolved in butan-1-ol (2 mL). Hydrazine hydrate (22 μL, 0.68 mmol) was added and heated in a microwave (150 °C, 1 h). Solvent was evaporated and the residue chromatographed (0 – 20% MeOH / DCM) to give **4** (37 mg, 0.095 mmol, 56%). ^{1}H NMR (DMSO-d_6): δ 12.62 (bs, 1H), 8.77 (bs, 1H), 7.33 – 7.22 (m, 6H), 4.30 (d, *J* = 13.4 Hz, 1H), 3.99 – 3.94 (m, 2H), 3.73 (d, *J* = 2.2 Hz, 2H), 3.16 – 3.12 (m, 1H), 2.82 – 2.76 (m ,1H), 2.65 (d, *J* = 7.2 Hz, 2H), 2.07 – 2.02 (m, 1H), 1.91 – 1.83 (m, 2H), 1.37 – 1.28 (m, 2H), 0.91 (d, *J* = 6.7 Hz, 6H); MS (ESI): m/z 393.2 [M + H]$^+$.

***N*-(1-(benzylsulfonyl)piperidin-4-yl)-3-*iso*butyl-1*H*-pyrazolo[3,4-*d*]pyrimidin-6-amine (5)**

51 (100 mg, 0.44 mmol) and DIPEA (92 μL, 0.48 mmol) in butan-1-ol (5 mL) were heated in a microwave (100 °C, 15 min) to give crude **55a**. To the RM was added hydrazine hydrate (28 μL,

1.76 mmol) and heated in a microwave (150 °C, 1 h). After cooling, Solvent was evaporated, water (5 mL) and DCM (5 mL) added and the organic layer separated, evaporated and purified by flash chromatography (0 – 20% MeOH / DCM) to give **5** (63 mg, 0.15 mmol, 34%). ^{1}H NMR (DMSO-d_6): δ 12.65 (bs, 1H), 8.79 (s, 1H), 7.49 – 7.35 (m, 6H), 4.42 (s, 2H), 3.81 (s, 1H), 3.56 (d, J = 12.4 Hz, 2H), 2.86 – 2.81 (m, 2H), 2.65 (d, J = 7.1 Hz, 2H), 2.07 – 2.02 (m, 1H), 1.93 – 1.91 (m, 2H), 1.52 – 1.45 (m, 2H), 0.91 (d, J = 6.6 Hz, 6H); MS (ESI): m/z 429.2 $[M + H]^+$.

1-(4-((3-*iso*Butyl-1*H*-pyrazolo[3,4-*d*]pyrimidin-6-yl)amino)piperidin-1-yl)-3-phenylethan-1-one (6)

6 was prepared by an analogous method to **4** starting from **54** (50 mg, 0.17 mmol) and 3-phenylpropanoic acid (28 mg, 19 mmol) to yield **6** (23 mg, 0.06 mmol, 33%). ^{1}H NMR (DMSO-d_6): δ 12.63 (bs, 1H), 8.78 (s, 1H), 7.28 – 7.18 (m, 6H), 4.32 – 4.30 (m, 2H), 4.00 – 3.85 (m, 2H), 3.15 – 3.06 (m, 1H), 2.85 – 2.73 (m, 2H), 2.69 – 2.62 (m, 4H), 2.09 – 2.02 (m, 1H), 1.93 – 1.86 (m, 2H), 1.44 – 1.31 (m, 2H), 0.92 (d, J = 6.5 Hz, 6H); MS (ESI): m/z 407.2 $[M + H]^+$.

(4-((3-*iso*Butyl-1*H*-pyrazolo[3,4-*d*]pyrimidin-6-yl)amino)piperidin-1-yl)(phenyl)methanone (7)

To **54** (50 mg, 0.17 mmol) and triethylamine (58 μL, 0.43 mmol) in DCM (3 mL) was added benzoyl chloride (24 μL, 0.20 mmol) and the mixture stirred overnight, then washed with water (3 ml). The organic layer was separated *via* a phase separator and the solvent evaporated. The crude mixture was dissolved in butan-1-ol (1 mL), hydrazine hydrate (30 μL, 0.93 mmol) added

and heated in a microwave (160 °C, 1 h). Solvent was evaporated and crude material chromatographed (0 – 20% MeOH / DCM) to give **7** (22 mg, 0.06 mmol, 35 %). ^{1}H NMR (DMSO-d_6): δ 12.64 (s, 1H), 8.78 (s, 1H), 7.48 – 7.45 (m, 3H), 7.40 – 7.37 (m, 3H), 4.344 – 4.37 (m, 1H), 4.08 – 3.99 (m, 1H), 3.67 – 3.57 (m, 1H), 3.21 – 3.13 (m, 1H), 3.05 – 2.98 (m, 1H), 2.64 (d, *J* = 7.15 Hz, 2H), 2.08 – 1.87 (m, 3H), 1.55 – 1.40 (m, 2H), 0.92 (d, *J* = 6.6 Hz, 6H). MS (ESI): *m/z* 379.2 $[M + H]^+$

2-(2,4-Difluorophenyl)-1-(4-((3-isobutyl-1*H*-pyrazolo[3,4-*d*]pyrimidin-6-yl)amino)piperidin-1-yl)ethan-1-one (8)

8 was prepared by an analogous method to **5**, starting from **51** (100 mg, 0.44 mmol) and 1-(4-aminopiperidin-1-yl)-2-(2,4-difluorophenyl)ethan-1-one (127 mg, 0.48 mmol) to give **8** (57 mg, 0.133 mmol, 30%. %). ^{1}H NMR (DMSO-d_6): δ 12.67 (bs, 1H), 8.79 (s, 1H), 7.33 (bs, 1H), 7.35 – 7.29 (m, 1H), 7.23 – 7.17 (m, 1H), 7.06 – 7.01 (m, 1H), 4.30 (d, *J* = 12.8 Hz, 1H), 3.99 (d, *J* = 13.9 Hz, 2H), 3.80 – 3.70 (m, 2H), 3.25 – 3.18 (m, 1H), 2.83 – 2.78 (m, 1H), 2.65 (d, *J* = 7.2 Hz, 2H), 2.08 – 2.01 (m, 1H), 1.98 – 1.88 (m, 2H), 1.50 – 1.32 (m, 2H), 0.91 (d, *J* = 6.6 Hz, 6H); MS (ESI): *m/z* 429.2 $[M + H]^+$

2-(2,4-Dichlorophenyl)-1-(4-((3-*iso*butyl-1*H*-pyrazolo[3,4-*d*]pyrimidin-6-yl)amino)piperidin-1-yl)ethan-1-one (9)

9 was prepared by an analogous method to **4** starting from **54** (50 mg, 0.17 mmol) and 2,4-dichlorophenylacetic acid (42 mg, 21 mmol) to yield **9** (24 mg, 0.05 mmol, 31%). ^{1}H NMR (DMSO-d_6): δ 12.64 (bs, 1H), 8.78 (s, 1H), 7.58 (d, *J* = 2.1 Hz, 1H), 7.38 – 7.33 (m, 3H), 4.23

(d, J = 12.7 Hz, 1H), 4.08 (d, J = 12.9 Hz, 2H), 3.89 – 3.77 (m, 2H), 3.24 – 3.20 (m, 1H), 2.82 – 2.78 (m, 1H), 2.64 (d, J = 7.2 Hz, 2H), 2.07 – 2.01 (m, 1H), 1.99 – 1.87 (m, 2H), 1.49 – 1.34 (m, 2H), 0.91 (d, J = 6.6 Hz, 6H); MS (ESI): *m/z* 461.2, 463.2 $[M + H]^+$

1-(4-((3-*iso*Butyl-1*H*-pyrazolo[3,4-*d*]pyrimidin-6-yl)amino)piperidin-1-yl)-2-phenylpropan-1-one (10)

10 was prepared by an analogous method to **4** starting from **54** (50 mg, 0.17 mmol) and 2-phenylpropionic acid (32 μL, 21 mmol) to yield **10** (28 mg, 0.07 mmol, 41%). ^{1}H NMR (DMSO-d_6): δ 12.64 (bs, 1H), 8.77 – 8.73 (m, 1H), 7.35 – 7.26 (m, 5H), 7.25 – 7.21 (m, 1H), 4.39 – 4.28 (m, 1H), 4.15 – 4.09 (m, 1H), 4.00 – 3.89 (m, 2H), 3.13 – 3.09 (m, 1H), 3.05 – 2.95 (m, 1H), 2.80 – 2.73 (m, 1H), 2.66 – 2.62 (m, 2H), 2.05 – 1.99 (m, 1H), 1.89 – 1.81 (m, 1H), 1.73 – 1.66 (m, 1H), 1.36 – 1.34 (m, 1H), 1.30 – 1.28 (m, 3H), 0.91 – 0.89 (m, 6H); MS (ESI): *m/z* 407.2 $[M + H]^+$

2-(2,4-Difluorophenyl)-1-(4-((3-neopentyl-1*H*-pyrazolo[3,4-*d*]pyrimidin-6-yl)amino)piperidin-1-yl)ethan-1-one (11)

To **57a** (50 mg, 0.16 mmol) in DMF (2 mL) was added DIPEA (68 μL, 0.32 mmol) and TBTU (68 mg, 0.21 mmol), stirred for 10 min then 2,4-difluorophenylacetic acid (34 mg, 0.21 mmol) added and stirred overnight. Water (5 mL) and DCM (5 mL) were added and the organic layer separated, washed with 20% aqueous NaCl, brine and the solvent evaporated. Crude material was taken up in butan-1-ol (2 mL), hydrazine hydrate (40 μL, 0.64 mmol) added and heated in a

microwave (150 °C, 1h). Solvent was evaporated, EtOH (3 mL) added and the resulting solid collected and dried to give **11** (31 mg, 0.07 mmol, 44%). ^{1}H NMR (DMSO-d_6): δ 12.73 (bs, 1H), 8.77 (s, 1H), 7.39 (bs, 1H), 7.37 – 7.29 (m, 1H), 7.22 – 7.17 (m, 1H), 7.06 – 7.01 (m, 1H), 4.29 (d, *J* = 13.1 Hz, 1H), 3.99 (d, *J* = 11.8 Hz, 2H), 3.79 – 3.70 (m, 2H), 3.24 – 3.19 (m, 1H), 2.83 – 2.79 (m, 1H), 2.67 (s, 2H), 2.00 – 1.90 (m, 2H), 1.49 – 1.32 (m, 2H), 0.95 (s, 9H); MS (ESI): *m/z* 443.2 $[M + H]^+$

1-(4-((3-Cyclopropyl-1*H*-pyrazolo[3,4-*d*]pyrimidin-6-yl)amino)piperidin-1-yl)-2-(2,4-difluorophenyl)ethan-1-one (12)

12 Was synthesised by an analogous method to **11** from **57b** (100 mg, 0.36 mmol) and 2,4-difluorophenylacetic acid (76 mg, 0.44 mmol) to yield **12** (46 mg, 0.11 mmol, 31%). ^{1}H NMR (DMSO-d_6): δ 12.43 (bs, 1H), 8.61 (s, 1H), 7.24 (bs, 1H), 7.19 – 7.14 (m, 1H), 7.07 – 7.02 (m, 1H), 6.91 – 6.86 (m, 1H), 4.13 (d, *J* = 13.9 Hz, 1H), 3.84 (d, *J* = 14.1 Hz, 2H), 3.63 – 3.55 (m, 2H), 3.07 – 3.03 (m, 1H), 2.68 – 2.61 (m, 1H), 2.05 – 2.00 (m, 1H), 1.77 – 1.73 (m, 2H), 1.33 – 1.17 (m, 2H), 0.85 – 0.77 (m, 4H); MS (ESI): *m/z* 443.2 $[M + H]^+$

2-(2,4-Difluorophenyl)-1-(4-((3-(tetrahydro-2*H*-pyran-4-yl)-1*H*-pyrazolo[3,4-*d*]pyrimidin-6-yl)amino)piperidin-1-yl)ethan-1-one (13)

To **59d** (195 mg, 0.41 mmol) in EtOH (5 mL) was added hydrazine hydrate (120 µL, 1.93 mmol) and heated in a sealed tube in a microwave (150 °C, 60 min). After cooling to RT, the resulting white precipitate was collected, washed with MeOH (5 mL) and dried to yield **13** (74 mg, 0.16 mmol, 39%). ^{1}H NMR (DMSO-d_6): δ 12.68 (br s, 1H), 8.88 (s, 1H), 7.48 – 7.24 (m, 2H), 7.18

(td, J = 9.8, 2.5 Hz, 1H), 7.03 (td, J = 8.5, 2.5 Hz, 1H), 4.28 (br d, J = 13.1 Hz, 1H), 4.07 – 3.87 (m, 4H), 3.74 (br d, J = 3.0 Hz, 2H), 3.55 – 3.40 (m, 2H), 3.27 – 3.09 (m, 2H), 2.81 (br t, J = 11.4 Hz, 1H), 2.08 – 1.76 (m, 6H), 1.58 – 1.22 (m, 2H). HRMS (ES^+): m/z $[M + H]^+$ calcd for $C_{23}H_{26}F_2N_6O_2$ 457.2163, found 457.2180.

2-(2,4-Difluorophenyl)-1-(4-((3-(morpholinomethyl)-1*H*-pyrazolo[3,4-*d*]pyrimidin-6-yl)amino)piperidin-1-yl)ethan-1-one (14)

To **56i** (780 mg, 2.5 mmol) and **58** (690 mg, 2.8 mmol) in EtOH (10 mL) was added DIPEA (516 µL, 3 mmol) and the mixture heated in a microwave (120 °C, 20 min). The solvent was evaporated and crude material purified by flash chromatography (0 – 2% MeOH / DCM) to yield **59i** (840 mg, 1.6 mmol, 63%) which was used without further purification. To crude **59i** (400 mg, 0.7 mmol) in THF (10 mL) was added TBAF (1M in THF, 750 µL, 0.7 mmol) drop-wise and stirred for 1 hour. The solution was diluted with DCM, washed with water and the solvent evaporated to yield crude **61** which was used in the next step without purification.

To **61** (70 mg, 0.17 mmol) in MeCN (5 mL) was added DIPEA (91 µL, 0.51 mmol) and methanesulfonyl chloride (15 µL, 0.19 mmol) and stirred for 2 h, after which time morpholine (15 µL, 0.34 mmol) was added and the RM heated in a microwave (100 °C, 0.5 h). Solvent was evaporated, MeOH (3 mL) added and the resulting solid collected and dried to yield **14** (15 mg, 0.031 mmol, 18%). ^1H NMR (DMSO-d_6): δ 12.88 (bs, 1H), 8.93 (s, 1H), 7.53 (bs, 1H), 7.40 – 7.35 (m, 1H), 7.29 – 7.23 (m, 1H), 7.12 – 7.07 (m, 1H), 4.35 (d, J = 13.9 Hz, 1H), 4.08 – 4.03 (m, 2H), 3.85 – 3.71 (m, 4H), 3.65 – 3.62 (m, 4H), 3.31 – 3.24 (m, 1H), 2.88 – 2.83 (m, 1H), 2.49 – 2.44 (m, 4H), 2.02 – 1.95 (m, 2H), 1.57 – 1.37 (m, 2H); MS (ESI): *m/z* 472.2 $[M + H]^+$

2-(2,4-Difluorophenyl)-1-(4-((3-morpholino-1*H*-pyrazolo[3,4-*d*]pyrimidin-6-yl)amino)piperidin-1-yl)ethan-1-one (15)

A solution of **71** (95 mg, 0.31 mmol), 1-hydroxybenzotriazole hydrate (62 mg, 0.41 mmol), 2,4-difluorophenylacetic acid (65 mg, 0.38 mmol), EDCI (78 mg, 0.41 mmol) and DIPEA (0.273 mL, 1.57 mmol) in DCM (5 mL) was stirred overnight at RT. Sat. aqueous NH_4Cl (10 mL) was added and extracted with DCM (2x10mL). The combined organics were dried over Na_2SO_4 and solvent evaporated to give crude product which was purified by flash chromatography (0 – 10% MeOH/DCM) to give **15** (52 mg, 0.114 mmol, 36%). ^{1}H NMR (DMSO-d_6): δ 12.02 (br s, 1H), 8.84 (s, 1H), 7.41 – 7.22 (m, 2H), 7.18 (td, *J* = 9.8 and 2.7 Hz, 1H), 7.06 – 6.98 (m, 1H), 4.35 – 4.19 (m, 1H), 4.04 – 3.90 (m, 2H), 3.80 – 3.68 (m, 6H), 3.31 – 3.27 (m, 4H), 3.24 – 3.16 (m, 1H), 2.84 – 2.75 (m, 1H), 1.98 – 1.85 (m, 2H), 1.51 – 1.28 (m, 2H). MS (ESI) m/z 458.3 $[M + H]^+$.

2-(2,4-Difluorophenyl)-1-(4-((3-(pyridin-2-yl)-1*H*-pyrazolo[3,4-*d*]pyrimidin-6-yl)amino)piperidin-1-yl)ethan-1-one (18)

18 was synthesised by an analogous method to **14**, using **56e** (100 mg, 0.4 mmol) and **58** (112 mg, 0.44 mmol) to yield **18** (48 mg, 0.11 mmol, 28%). ^{1}H NMR (DMSO-d_6): δ 13.35 (s, 1H), 9.37 (s, 1H), 8.71 (d, *J* = 4.8 Hz, 1H), 8.09 (d, *J* = 8.0 Hz, 1H), 7.93 – 7.89 (m, 1H), 7.67 (bs, 1H), 7.44 – 7.40 (m, 1H), 7.35 – 7.30 (m, 1H), 7.23 – 7.18 (m, 1H), 7.06 – 7.02 (m, 1H), 4.30 (d, *J* = 14.1 Hz, 1H), 4.02 (d, *J* = 12.8 Hz, 2H), 3.81 – 3.71 (m, 2H), 3.26 – 3.22 (m, 1H), 2.86 – 2.81 (m, 1H), 2.00 – 1.94 (m, 2H), 1.52 – 1.36 (m, 2H); MS (ESI): *m/z* 450.2 $[M + H]^+$

2-(2,4-Difluorophenyl)-1-(4-((3-(1-methyl-1*H*-pyrazol-5-yl)-1*H*-pyrazolo[3,4-*d*]pyrimidin-6-yl)amino)piperidin-1-yl)ethan-1-one (19)

A mixture of **56f** (80 mg,0.32 mmol), **58** (89 mg,0.35 mmol) and DIPEA (66 µl, 0.38 mmol) in EtOH (2 mL) was heated in a microwave (120 °C, 30 min). Upon cooling, hydrazine hydrate (75 µl, 1.28 mmol) was added and the mixture further heated in a microwave (150 °C, 1 h). Upon cooling, solvent was evaporated and crude material purified by preparative HPLC to yield **19** (45 mg, 0.1 mmol, 31 %). ^{1}H NMR (DMSO-d_6): δ 13.38 (s, 1H), 9.03 (s, 1H), 7.68 – 7.61 (m, 1H), 7.55 (d, *J* = 7.6 Hz, 1H), 7.32 (dd, *J* = 15.3, 8.4 Hz, 1H), 7.22 – 7.16 (m, 1H), 7.06 – 6.96 (m, 2H), 4.31 (d, *J* = 13.1 Hz, 1H), 4.13 (s, 3H), 4.02 (d, *J* = 12.3 Hz, 2H), 3.76 (dd, *J* = 16.3, 23.5 Hz, 2H), 3.28 – 3.19 (m, 1H), 2.86 – 2.78 (m, 1H), 2.01 – 1.90 (m, 2H), 1.55 – 1.33 (m, 2H). MS (ESI): *m/z* 453.0 $[M + H]^+$

2-(2,4-Difluorophenyl)-1-(4-((3-(2-methoxyphenyl)-1*H*-pyrazolo[3,4-*d*]pyrimidin-6-yl)amino)piperidin-1-yl)ethan-1-one (20)

To a solution of **56c** (278 mg, 1 mmol) and **58** (280 mg, 1.1 mmol) in EtOH (5 mL) was added DIPEA (209 µL, 2 mmol) and the mixture heated in a microwave (120 °C, 30 min). After cooling, hydrazine hydrate (300 µl, 4 mmol) was added and the mixture heated in a microwave (150 °C, 1 hour). Solvent was evaporated, MeCN (5 mL) added and filtered to remove the solid. The solution was allowed to stand overnight, after which time a suspension formed which was collected, washed with further MeCN and dried to yield **20** (355 mg, 0.74 mmol, 74%). ^{1}H NMR (DMSO-d_6): δ 13.17 (s, 1H), 8.79 (s, 1H), 7.67 (dd, *J* = 1.7, 7.6 Hz, 1H), 7.44 (m, 2H), 7.33 (m,

1H), 7.20 (m, 2H), 7.04 (m, 2H), 4.30 (d, *J* = 13.0 Hz, 1H), 4.00 (d, *J* = 11.8 Hz, 2H), 3.87 (s, 3H), 3.81 – 3.71 (m, 2H), 3.24 (t, *J* = 11.7 Hz, 1H), 2.82 (t, *J* = 11.5 Hz, 1H), 1.97 (m, 2H), 1.51 – 1.35 (m, 2H). MS (ESI): *m/z* 479.1 $[M + H]^+$.

2-(2,4-Difluorophenyl)-1-(4-((3-(2-methoxypyridin-3-yl)-1*H*-pyrazolo[3,4-*d*]pyrimidin-6-yl)amino)piperidin-1-yl)ethan-1-one (21)

21 Was prepared by an analogous method to **20** from **56h** (114 mg, 0.41 mmol) and **58** (100 mg, 0.45 mmol), yielding **21** (28 mg, 0.06 mmol, 13%). ^{1}H NMR (DMSO-d_6): δ 13.32 (s, 1H), 8.90 (s, 1H), 8.28 (dd, *J* = 1.9, 4.9 Hz, 1H), 8.10, (dd, *J* = 1.7, 7.3 Hz, 1H), 7.51 (s, 1H), 7.35 – 7.30 (m, 1H), 7.23 – 7.18 (m, 1H), 7.14 (dd, *J* = 4.9, 7.5 Hz, 1H), 7.06 – 7.02 (m, 1H), 4.30 (d, *J* = 12.6 Hz, 1H), 4.05 – 3.97 (m, 5H), 3.77 (dd, *J* = 16, 27 Hz, 2H), 3.26 – 3.20 (m, 1H), 2.85 – 2.78 (m, 1H), 2.02 – 1.91 (m, 2H), 1.49 – 1.38 (m, 2H). MS (ESI): *m/z* 480.2 $[M + H]^+$.

2-(2,4-Difluorophenyl)-1-(4-((3-*iso*butyl-1*H*-pyrazolo[3,4-*d*]pyrimidin-6-yl)(methyl)amino)piperidin-1-yl)ethan-1-one (22)

To **51** (80 mg, 0.35 mmol) and **63a** (83 mg, 0.39 mmol) in butan-1-ol (1 mL) was added DIPEA (0.73 mL, 0.42 mmol) and heated in a microwave (120 °C, 30 min). Solvent was evaporated and crude material taken up in DCM (2 mL) / TFA (2 mL) and stirred overnight. Solvent was evaporated and crude material taken up in MeOH (1 mL) and passed through an SCX column, eluting with 2M NH_3 / MeOH. After evaporation of solvent, the free-base was taken up in DCM (2 mL) and 2-(2,4-difluorophenyl)acetic acid (66 mg, 0.42 mmol), TBTU (146 mg, 0.46 mmol)

and DIPEA (146 ml, 0.7 mmol) added and stirred for 2 h. The mixture was washed with water (2 mL) and the organic layer evaporated, taken up in butan-1-ol (3 mL), hydrazine hydrate (0.60 mL, 1.4 mmol) added and heated in a microwave (160 °C, 1 h). After cooling, solvent was evaporated and crude material chromatographed (0 – 20% MeOH / DCM) to yield **22** (44 mg, 0.1 mmol, 28%). ^{1}H NMR (DMSO-d_6): δ 12.71 (s, 1H), 8.88 (s, 1H), 7.37 – 7.30 (m, 1H), 7.23 – 7.17 (m, 1H), 7.04 (ddd, *J* = 8.5 , 8.5 and 2.3 Hz, 1H), 4.95 (s, 1H), 4.52 (d, *J* = 13.2 Hz, 1H), 4.13 (d, *J* = 13.5 Hz, 1H), 3.82 – 3.73 (m, 2H), 3.22 – 3.16 (m, 1H), 3.00 (s, 3H), 2.71 – 2.65 (m, 3H), 2.11 – 2.02 (m, 1H), 1.76 – 1.59 (m, 4H), 0.90 (d, *J* = 6.7 Hz, 6H); MS (ESI): *m/z* 443.2 $[M + H]^+$.

2-(2,4-Difluorophenyl)-1-(4-((3-*iso*butyl-1-methyl-1*H*-pyrazolo[3,4-*d*]pyrimidin-6-yl)amino)piperidin-1-yl)ethan-1-one (24)

To **51** (100 mg, 0.44 mmol) and **58** (123 mg, 0.48 mmol) in butan-1-ol (5 mL) was added DIPEA (93 mL, 0.53 mmol) and the RM heated in a microwave (110 °C, 20 min). After cooling, methylhydrazine (96 µL, 1.76 mmol) was added and the RM heated in a microwave (150 °C, 45 min). Solvent was evaporated, MeCN (3 mL) added and the resulting solid collected, washed with MeCN and dried to give **24** (35 mg, 0.079 mmol, 18%). ^{1}H NMR (DMSO-d_6): δ 8.78 (bs, 1H), 7.42 (bs, 1H), 7.25 – 7.29 (m, 1H), 7.21 – 7.16 (m, 1H), 7.05 – 7.01 (m, 1H), 4.27 (d, *J* = 12.7 Hz, 1H), 4.12 – 4.06 (m, 1H), 4.00 – 3.96 (m, 1H), 3.79 – 3.70 (m, 5H), 3.28 – 3.22 (m, 1H), 2.89 – 2.82 (m, 1H), 2.66 (d, *J* = 7.2 Hz, 2H), 2.08 – 1.91 (m, 3H), 1.51 – 1.35 (m, 2H), 0.92 (d, *J* = 6.6 Hz, 6H); MS (ESI): m/z 443.2 $[M + H]^+$.

2-(2,4-Difluorophenyl)-1-(4-((5-(2-methoxyphenyl)-7*H*-pyrrolo[2,3-*d*]pyrimidin-2-yl)amino)piperidin-1-yl)ethan-1-one (26)

Crude **67** (130 mg, 0.36 mmol), **58** (184 mg, 0.72 mmol) and DIPEA (195 µl, 1.08 mmol) in NMP (2 mL) were heated in a microwave (250 °C, 30 min). The reaction mixture was poured into 20% aqueous NaCl (15 mL) and extracted with EtOAc (15 mL x 3). The combined organics were washed with brine (25 mL), dried, and the solvent evaporated. MeCN (5 ml) was added and the resulting solid collected, washed with further MeCN and dried to give **26** (30 mg, 0.63 mmol, 17%). ^{1}H NMR (DMSO-d_6): δ 11.50 (s, 1H), 8.72 (s, 1H), 8.09 (d, *J* = 7.8 Hz, 1H), 7.55 (dd, *J* = 7.5, 1.7 Hz, 1H), 7.39 – 7.35 (m, 2H), 7.29 – 7.25 (m, 1H), 7.21 – 7.17 (ddd, *J* = 10.0, 9.6, 2.6 Hz, 1H), 7.11 (d, *J* = 7.6 Hz, 1H), 7.05 – 7.00 (m, 2H), 4.57 (d, *J* = 12.9 Hz, 2H), 3.89 – 3.82 (m, 4H), 3.45 (s, 2H), 3.10 – 3.04 (m, 2H), 1.84 – 1.79 (m, 2H), 1.42 – 1.32 (m, 2H). MS (ESI): *m/z* 478.1 $[M + H]^+$.

1-(4-((4-Amino-5-(2-methoxybenzoyl)pyrimidin-2-yl)amino)piperidin-1-yl)-2-(2,4-difluorophenyl)ethan-1-one (27)

A solution of **64k** (100 mg, 0.20 mmol) in EtOH (1 mL) / ammonium hydroxide (1 mL) was heated in a microwave (140 °C, 30 min). Upon cooling, the resulting solid was collected, washed with a mixture of water / EtOH (1:1) and dried under vacuum to give **27** (80 mg, 0.17 mmol, 83%). ^{1}H NMR ($CDCl_3$): δ 8.17 - 8.05 (m, 1H), 7.47 – 7.42 (m, 1H), 7.33 – 7.25 (m, 2H, under solvent peak), 7.09 – 6.98 (m, 2H), 6.90 – 6.82 (m, 2H), 4.54 (d, *J* = 12.5 Hz, 1H), 4.11 (bs, 1H), 3.93 – 3.88 (m, 1H), 3.82 (s, 3H), 3.77 – 3.67 (m, 3H), 3.28 – 3.20 (m, 1H), 2.94 – 2.87 (m, 1H), 2.11 – 2.06 (m, 2H), 1.57 (s, 2H), 1.46 – 1.29 (m, 2H); MS (ESI): *m/z* 481.9 $[M + H]^+$

(*S*)-2-(2,4-Difluorophenyl)-1-(3-((3-(pyridin-2-yl)-1*H*-pyrazolo[3,4-*d*]pyrimidin-6-yl)amino)piperidin-1-yl)ethan-1-one (29)

29 Was prepared by an analogous method to **22** from **56e** (300 mg, 1.2 mmol), **63g** (265 mg, 1.4 mmol) and DIPEA (252 μL, 1.4 mmol), then 2-(2,4-difluorophenyl)acetic acid (30 mg, 0.17 mmol), TBTU (62 mg, 0.18 mmol) and DIPEA (62 μL, 0.34 mmol) to yield **29** (28 mg, 0.06 mmol, 33% over 2 steps). ^{1}H NMR (DMSO-d_6): δ 13.35 (s, 1H), 9.39 (d, *J* = 17.7 Hz, 1H), 8.73 – 8.70 (m, 1H), 8.11 – 8.07 (m, 1H), 7.93 – 7.89 (m, 1H), 7.69 – 7.57 (m, 1H), 7.44 – 7.39 (m, 1H), 7.35 – 7.25 (m, 1H), 7.23 – 7.12 (m, 1H), 7.07 – 6.99 (m, 1H), 4.40 – 4.06 (m, 1H), 3.90 – 3.69 (m, 4H), 3.24 – 2.67 (m, 2H), 2.05 – 1.99 (m, 1H), 1.86 – 1.77 (m, 1H), 1.68 – 1.40 (m, 2H). MS (ESI): *m/z* 450.1[M + H]$^+$.

(*R*)-2-(2,4-Difluorophenyl)-1-(3-((3-(pyridin-2-yl)-1*H*-pyrazolo[3,4-*d*]pyrimidin-6-yl)amino)piperidin-1-yl)ethan-1-one (30)

30 Was prepared by an analogous method to **22** from **56e** (300 mg, 1.2 mmol), **63h** (265 mg, 1.4 mmol) and DIPEA (252 μL, 1.4 mmol), then 2-(2,4-difluorophenyl)acetic acid (30 mg, 0.17 mmol), TBTU (62 mg, 0.18 mmol) and DIPEA (62 μL, 0.34 mmol) to yield **30** (36 mg, 0.08 mmol, 43% over 2 steps). ^{1}H NMR (DMSO-d_6): δ 13.34 (s, 1H), 9.37 (d, *J* = 18.1 Hz, 1H), 8.72 – 8.69 (m, 1H), 8.10 – 8.06 (m, 1H), 7.92 – 7.88 (m, 1H), 7.73 – 7.55 (m, 1H), 7.43 – 7.38 (m, 1H), 7.35 – 7.24 (m, 1H), 7.23 – 7.12 (m, 1H), 7.07 – 6.98 (m, 1H), 4.41 – 4.03 (m, 1H), 3.90 – 3.68 (m, 4H), 3.22 – 2.66 (m, 2H), 2.04 – 1.98 (m, 1H), 1.85 – 1.75 (m, 1H), 1.69 – 1.37 (m, 2H); MS (ESI): m/z 450.1[M + H]$^+$.

2-(2,4-Difluorophenyl)-1-(4-(((3-(pyridin-2-yl)-1*H*-pyrazolo[3,4-*d*]pyrimidin-6-yl)amino)methyl)piperidin-1-yl)ethan-1-one (34)

34 Was prepared by an analogous method to **22** from **56e** (500 mg, 2.0 mmol), **63i** (429 mg, 2 mmol) and DIPEA (419 µL, 2.4 mmol), then 2-(2,4-difluorophenyl)acetic acid (28 mg, 0.17 mmol), TBTU (59 mg, 0.18 mol) and DIPEA (59 µL, 0.39 mmol) to yield **34** (25 mg, 0.05 mmol, 15% over 2 steps). ^{1}H NMR (DMSO-d_6): δ 13.30 (s, 1H), 9.34 (s, 1H), 8.71 (d, *J* = 4.7 Hz, 1H), 8.07 (d, *J* = 8.0 Hz, 1H), 7.91 (ddd, *J* = 7.7, 7.7 and 1.7 Hz, 1H), 7.70 (s, 1H), 7.44 – 7.39 (m, 1H), 7.30 (dd, *J* = 8.5, 7.3 Hz, 1H), 7.18 (ddd, *J* = 10.1, 9.8 and 2.6 Hz, 1H), 7.03 (ddd, *J* = 8.8, 8.5 and 2.7 Hz, 1H), 4.35 (d, *J* = 13.2 Hz, 1H), 4.00 (d, *J* = 14.9 Hz, 1H), 3.70 (dd, *J* = 16.4, 23.8 Hz, 2H), 3.28 – 3.20 (m, 2H), 3.08 – 3.02 (m, 1H), 2.67 -2.55 (m, 1H, under DMSO signal), 1.98 – 1.88 (m, 1H), 1.82 – 1.72 (m, 2H), 1.20-1.00 (m, 2H). MS (ESI): *m/z* 464.1 [M + H]$^+$.

2-(2,4-Difluorophenyl)-1-(3-(((3-(2-methoxyphenyl)-1*H*-pyrazolo[3,4-*d*]pyrimidin-6-yl)amino)methyl)azetidin-1-yl)ethan-1-one (35)

35 Was prepared by an analogous method to **22** from **56c** (300 mg, 1 mmol), **63f** (220 mg, 1.1.mmol) and DIPEA (225 µL, 1.3 mmol) then 2-(2,4-difluorophenyl)acetic acid (28 mg, 0.17 mmol), TBTU (59 mg, 0.18 mol) and DIPEA (59 µL, 0.39 mmol) to yield **35** (10 mg, 0.022 mmol, 14%). ^{1}H NMR (DMSO-d_6): δ 13.17 (s, 1H), 8.78 (s, 1H), 7.68 – 7.63 (m, 2H), 7.44 (m, 1H), 7.33 (m, 1H), 7.20 – 7.16 (m, 2H), 7.07 – 7.01 (m, 2H), 4.27 (dd, *J* = 8.5, 8.5 Hz, 1H), 3.98

– 3.91 (m, 2H), 3.87 (s, 3H), 3.69 – 3.65 (m, 1H), 3.59 – 3.54 (m, 2H), 3.44 (s, 2H), 2.95 – 2.89 (m, 1H); MS (ESI): *m/z* 465.0 $[M + H]^+$.

2-(2,4-Difluorophenyl)-*N*-(2-methyl-2-((3-(pyridin-2-yl)-1*H*-pyrazolo[3,4-*d*]pyrimidin-6-yl)amino)propyl)acetamide (36)

36 Was prepared by an analogous method to **22** from **56e** (500 mg, 2.0 mmol), **63j** (377 mg, 2 mmol) and DIPEA (419 µL, 2.4 mmol) then 2-(2,4-difluorophenyl)acetic acid (28 mg, 0.17 mmol), TBTU (59 mg, 0.18 mol) and DIPEA (59 µL, 0.39 mmol) to yield **36** (10 mg, 0.022 mmol, 15% over 2 steps). ^{1}H NMR (DMSO-d_6): δ 13.3 (s, 1H), 9.34 (s, 1H), 8.73 – 8.71 (m, 1H), 8.17 (dd, *J* = 8.2, 8.2 Hz, 1H), 8.10 (d, *J* = 8.0 Hz, 1H), 7.91 (ddd, *J* = 7.9, 7.7 and 1.8 Hz, 1H), 7.42 – 7.35 (m, 2H), 7.18 (ddd, *J* = 9.8, 9.8 and 2.6 Hz, 1H), 7.06 – 7.00 (m, 2H), 3.56 – 3.51 (m, 4H), 1.39 (s, 6H); MS (ESI): *m/z* 438.1 $[M + H]^+$.

Ethyl 4-((3-(2-methoxyphenyl)-1*H*-pyrazolo[3,4-*d*]pyrimidin-6-yl)amino)piperidine-1-carboxylate (38)

56c (100 mg, 0.36 mmol), 1-(ethoxycarbonyl)-4-aminopiperidine (68 mg, 0.39 mmol) and DIPEA (79 µL, 0.43 mmol) in EtOH (2 mL) were heated in a microwave (120 °C, 30 min) to give crude **62c** (R^2 = ethoxycarbonyl). After cooling, hydrazine hydrate (200 µL, 0.36 mmol) was added and the RM heated in a microwave (120 °C, 1 h) then cooled to RT. The resulting solid was collected, washed with EtOH then water and dried to yield **38** (75 mg, 0.19 mmol, 53%). ^{1}H NMR (DMSO-d_6): δ 13.15 (bs, 1H), 8.78 (s, 1H), 7.66 (dd, *J* = 1.7 and 7.6 Hz, 1H),

7.46 – 7.42 (m, 1H), 7.38 (s, 1H), 7.19 (d, *J* = 8.3 Hz, 1H), 7.07 – 7.03 (m, 1H), 4.05 (q, *J* = 7.1 Hz, 2H), 4.01 – 3.94 (m, 3H), 3.86 (s, 3H), 3.02 – 2.90 (m, 2H), 1.95 – 1.89 (m, 2H), 1.46 – 1.37 (m, 2H), 1.20 (t, *J* = 7.1 Hz, 3H); MS (ESI): *m/z* 397.1 $[M + H]^+$.

Benzyl 4-((3-(2-methoxyphenyl)-1*H*-pyrazolo[3,4-*d*]pyrimidin-6-yl)amino)piperidine-1-carboxylate (39)

39 was prepared by an analogous method to **38** from **56c** (100 mg, 0.36 mmol), 1-(benzyloxycarbonyl)-4-aminopiperidine (92 mg, 0.39 mmol), DIPEA (79 µL, 0.43 mmol) and hydrazine hydrate (200 µL, 0.36 mmol) to yield **39** (20 mg, 0.04 mmol, 12%). ^{1}H NMR (DMSO-*d*$_6$): δ 13.17 (s, 1H), 8.78 (s, 1H), 7.66 (d, *J* = 6.4 Hz, 1H), 7.46 – 7.32 (m, 7H), 7.19 (d, *J* = 8.4 Hz, 1H), 7.08 – 7.04 (m, 1H), 5.10 (s, 2H), 4.08 – 3.94 (m, 3H), 3.86 (s, 3H), 3.08 – 2.90 (m, 2H), 1.96 – 1.90 (m, 2H), 1.48 – 1.38 (m, 2H); MS (ESI): *m/z* 459.1 $[M + H]^+$.

(4-((3-(2-Methoxyphenyl)-1*H*-pyrazolo[3,4-*d*]pyrimidin-6-yl)amino)piperidin-1-yl)(morpholino)methanone (40)

40 Was prepared by an analogous method to **11** from **57c** (515 mg, 1.5 mmol), morpholine-4-carbonyl chloride (191µl, 1.7 mmol) and DIPEA (315 µL, 1.9 mmol) then hydrazine hydrate (347 µL, 6 mmol) to yield **40** (270 mg, 0.6 mmol, 41 %). ^{1}H NMR (DMSO-*d*$_6$): δ 13.11 (s, 1H), 8.78 (s, 1H), 7.67 (dd, *J* = 7.5, 1.7 Hz, 1H), 7.46 – 7.42 (m, 1H), 7.33 (s, 1H), 7.18 (d, *J* = 8.0 Hz, 1H), 7.06 (ddd, *J* = 8.3, 7.5, 0.9 Hz, 1H), 3.99 – 3.93 (m, 1H), 3.87 (s, 3H), 3.63 (d, *J* = 13.2

Hz, 2H), 3.60 – 3.58 (m, 4H), 3.15 – 3.12 (m, 4H), 2.92 – 2.86 (m, 2H), 1.90 (d, *J* = 11.1 Hz, 2H), 1.52 – 1.44 (m, 2H). MS (ESI): *m/z* 438.0 $[M + H]^+$.

2-Cyclopropyl-1-(4-((3-(2-methoxyphenyl)-1*H*-pyrazolo[3,4-*d*]pyrimidin-6-yl)amino)piperidin-1-yl)ethan-1-one (42)

42 Was prepared by an analogous method to **11** from **57c** (2 g, 5.8 mmol) and 2-cyclopropylacetic acid (643 mg, 6.4 mmol) then hydrazine hydrate (2.7 ml, 87 mmol) to yield **42** (1.2 g, 2.8 mmol, 48 %). ^{1}H NMR (DMSO- d_6): δ 13.12 (s, 1H), 8.79 (s, 1H), 7.68 (dd, *J* = 7.6, 1.7 Hz, 1H), 7.48 – 7.45 (m, 1H), 7.34 (bs, 1H), 7.20 (d, *J* = 8.0 Hz, 1H), 7.06 (ddd, *J* = 7.5, 7. and 0.9 Hz, 1H), 4.40 – 4.35 (m, 1H), 4.10 – 4.01 (m, 1H), 3.92 – 3.87 (m, 4H), 3.21 – 3.15 (m, 1H), 2.82 – 2.76 (m, 1H), 2.33 – 2.30 (m, 2H), 2.02 – 1.93 (m, 2H), 1.50 – 1.34 (m, 2H), 1.03 – 0.97 (m, 1H), 0.52 – 0.48 (m, 2H), 0.19 – 0.15 (m, 2H).

2-(3,4-Difluorophenyl)-1-(4-((3-(2-methoxyphenyl)-1*H*-pyrazolo[3,4-*d*]pyrimidin-6-yl)amino)piperidin-1-yl)ethan-1-one (43)

43 Was prepared by an analogous method to **11** from **57c** (100 mg, 0.29 mmol), and (3,4-Difluorophenyl)acetic acid (44 mg, 0.32 mmol) then hydrazine hydrate (137 μL, 4.4 mmol) to yield **43** (30 mg, 0.06 mmol, 22%). ^{1}H NMR (DMSO- d_6): δ. 13.15 (s, 1H), 8.81 (s, 1H), 7.66 (dd, *J* = 1.6 and 7.6 Hz, 1H), 7.46 – 7.29 (m, 4H), 7.19 (d, *J* = 8.2 Hz, 1H), 7.11 – 7.08 (m, 1H), 7.07 – 7.04 (m, 1H), 4.33 (d, *J* = 13.4 Hz, 1H), 4.00 (d, *J* = 13.1 Hz, 2H), 3.87 (s, 3H), 3.77 (s, 2H), 3.22 – 3.15 (m, 1H), 2.84 – 2.77 (m, 1H), 1.96 – 1.91 (m, 2H), 1.41 – 1.31 (m, 2H).

2-Cyclopropyl-1-(4-((3-(tetrahydro-2*H*-pyran-4-yl)-1*H*-pyrazolo[3,4-*d*]pyrimidin-6-yl)amino)piperidin-1-yl)ethan-1-one (44)

44 Was prepared by an analogous method to **11** from **57d (**100 mg, 0.31 mmol) and 2-cyclopropylacetic acid (34 mg, 0.34 mmol) then hydrazine hydrate (80 µL, 1.24 mmol) to yield **44** (60 mg, 0.16 mmol, 50%). ^{1}H NMR (DMSO-d_6): δ 12.55 (s, 1H), 8.75 (s, 1H), 7.20 (bs, 1H), 4.19 (d, *J* = 12.5 Hz, 1H), 3.91 – 3.67 (m, 3H), 3.41 – 3.21 (m, 3H), 3.08 – 2.94 (m, 2H), 2.67 – 2.56 (m, 1H), 2.16 – 2.12 (m, 2H), 1.85 – 1.60 (m, 6H), 1.35 – 1.14 (m, 2H), 0.90 – 0.77 (m, 1H), 0.36 – 0.29 (m, 2H), 0.02 – -0.03 (m, 2H); MS (ESI): m/z 385.1 $[M + H]^+$.

2-Cyclopropyl-1-(4-((3-(pyridin-2-yl)-1*H*-pyrazolo[3,4-*d*]pyrimidin-6-yl)amino)piperidin-1-yl)ethan-1-one (45)

45 was prepared by an analogous method to **38** from **56e** (250 mg, 1 mmol), 1-(4-Amino-piperidin-1-yl)-2-cyclopropyl-ethanone (200 mg, 1.1 mmol) and DIPEA (208 µL, 1.2 mmol) then hydrazine hydrate (230 µL, 4 mmol) to yield **45 (**85 mg, 0.23 mmol, 23%). ^{1}H NMR (DMSO-d_6): δ 13.18 (s, 1H), 9.22 (s, 1H), 8.59 – 8.57 (m, 1H), 7.96 – 7.94 (m, 1H), 7.77 (dt, *J* = 1.8 and 7.8 Hz, 1H), 7.44 (bs, 1H), 7.29 – 7.26 (m, 1H), 4.20 (d, *J* = 13.1 Hz, 1H), 3.91 – 3.83 (m, 1H), 3.73 (d, *J* = 13.7 Hz, 1H), 3.05 – 2.97 (m, 1H), 2.67 – 2.60 (m, 1H), 2.16 – 2.13 (m, 2H), 1.86 – 1.77 (m, 2H), 1.37 – 1.20 (m, 2H), 0.87 – 0.80 (m, 1H), 0.35 – 0.31 (m, 2H), 0.02 – -0.02 (m, 2H); MS (ESI): m/z 378.0 $[M + H]^+$.

2-Cyclopropyl-1-(4-((3-morpholino-1*H*-pyrazolo[3,4-*d*]pyrimidin-6-yl)amino)piperidin-1-yl)ethan-1-one (46)

To a solution of cyclopropylacetic acid (31 mg, 0.31 mmol) in DMF (1 mL) was added DIPEA (0.22 mL, 1.29 mmol) and TBTU (99 mg, 0.31 mmol) and the mixture stirred at RT for 30 min. **71** (80 mg, 0.24 mmol) was added portion wise and the mixture stirred for 1 h at RT. Sat. aqueous NH_4Cl was added and the aqueous layer extracted with EtOAc. The organic phase was dried over Na_2SO_4 and solvent evaporated to give crude product which was purified by preparative HPLC to give **46** (36 mg, 0.09 mmol, 38.9%). ^{1}H NMR (DMSO-d_6): δ 12.13 – 11.94 (m, 1H), 8.85 (s, 1H), 7.43 – 7.18 (m, 1H), 4.32 (br d, *J* = 13.8 Hz, 1H), 4.03 – 3.91 (m, 1H), 3.85 (br d, *J* = 13.8 Hz, 1H), 3.79 – 3.72 (m, 4H), 3.31 – 3.29 (m, 4H), 3.12 (br t, *J* = 11.7 Hz, 1H), 2.81 – 2.69 (m, 1H), 2.27 (dd, *J* = 6.8, 3.0 Hz, 2H), 1.98 – 1.84 (m, 2H), 1.50 – 1.26 (m, 2H), 1.04 – 0.89 (m, 1H), 0.50 – 0.42 (m, 2H), 0.17 – 0.09 (m, 2H).

2-(3,4-Difluorophenyl)-1-(4-((3-(tetrahydro-2*H*-pyran-4-yl)-1*H*-pyrazolo[3,4-*d*]pyrimidin-6-yl)amino)piperidin-1-yl)ethan-1-one (47)

47 Was prepared by an analogous method to **11** from **57d** (100 mg, 0.31 mmol) and 3,4-difluorophenylacetic acid (65 mg, 0.37 mmol), then hydrazine hydrate (119 μL, 3.8 mmol) to yield **47** (60 mg, 0.13 mmol, 42%). ^{1}H NMR (DMSO-d_6): δ 12.68 (s, 1H), 8.89 (s, 1H), 7.41 – 7.28 (m, 3H), 7.11 – 7.07 (m, 1H), 4.29 (d, *J* = 13.4 Hz, 1H), 4.00 – 3.92 (m, 4H), 3.75 (s, 2H), 3.52 – 3.45 (m, 2H), 3.20 – 3.13 (m, 2H), 2.83 – 2.77 (m, 1H), 1.93 – 1.77 (m, 6H), 1.40 – 1.30 (m, 2H); MS (ESI): m/z 456.9 $[M + H]^+$

2-(3,4-Difluorophenyl)-1-(4-((3-morpholino-1*H*-pyrazolo[3,4-*d*]pyrimidin-6-yl)amino)piperidin-1-yl)ethan-1-one (48)

To a solution of **71** (0.9 g, 2.65 mmol) and 3,4-difluorophenylacetic acid (0.547 g, 3.18 mmol) in DMF (20 mL) was added DIPEA (1.850 mL, 10.59 mmol), stirred for 5 min and HBTU (1.205 g, 3.18 mmol) added portion-wise. The mixture was stirred at RT for 1 h, 3,4-difluorophenylacetic acid (0.137 g, 0.80 mmol) added and the mixture stirred for 1 h. Sat'd aqueous NH_4Cl (40 mL) was added and the aqueous layer extracted with EtOAc (3 x 100 mL). The combined organics were dried over Na_2SO_4 and solvent evaporated to give crude product which was purified by flash chromatography (0 – 50% EtOAc / cyclohexane). The resulting solid was triturated with ether to give **48** (520 mg, 1.137 mmol, 43 %). ^{1}H NMR (DMSO-d_6): δ 12.11 – 11.90 (m, 1H), 8.83 (s, 1H), 7.41 – 7.23 (m, 3H), 7.12 – 7.02 (m, 1H), 4.35 – 4.21 (m, 1H), 4.02 – 3.87 (m, 2H), 3.78 – 3.70 (m, 6H), 3.32 – 3.25 (m, 4H), 3.14 (br t, *J* = 11.5 Hz, 1H), 2.77 (br t, *J* = 11.5 Hz, 1H), 1.93 – 1.81 (m, 2H), 1.41 – 1.23 (m, 2H). MS (ESI): m/z 458 $[M + H]^+$.

***N*-(1-(6-Chloropyridazin-3-yl)piperidin-4-yl)-3-morpholino-1*H*-pyrazolo[3,4-*d*]pyrimidin-6-amine (50)**

To a solution of **71** (1.0 g, 2.94 mmol) in EtOH (15 mL) was added DIPEA (2.056 mL, 11.77 mmol) and 3,6-dichloropyridazine (2.192 g, 14.71 mmol and the resulting mixture stirred at 130 ºC for 45 min (microwave). The resulting solid was washed with EtOH and hot MeOH, dissolved in HCl (1 M) and washed with DCM. The aqueous layer was treated with NaOH (2 M) until a precipitate appeared and this solid was filtered and washed with water to give **50** (440 mg, 1.058

mmol, 36 %). ^{1}H NMR (DMSO-d_6): δ 12.15 – 11.92 (m, 1H), 8.90 – 8.75 (m, 1H), 7.56 – 7.46 (m, 1H), 7.46 – 7.38 (m, 1H), 7.37 – 7.17 (m, 1H), 4.37 – 4.23 (m, 2H), 4.14 – 3.92 (m, 1H), 3.80 – 3.68 (m, 4H), 3.32 – 3.26 (m, 4H), 3.10 (br t, *J* = 11.5 Hz, 2H), 2.02 – 1.89 (m, 2H), 1.57 – 1.41 (m, 2H). MS (ESI): m/z 416.0 $[M + H]^+$.

1-(4-Methoxy-2-(piperidin-4-ylamino)pyrimidin-5-yl)-3-methylbutan-1-one (54)

To **51** (720 mg, 3.2 mmol) and **52** (695 mg, 3.5 mmol) in butan-1-ol (5 mL) was added DIPEA (660 mL, 3.8 mmol) and the mixture heated in a microwave (120 °C, 30 min). Solvent was evaporated and crude material taken up in DCM (2 mL) / TFA (2 mL) and stirred overnight. Solvent was evaporated and crude material taken up in MeOH and passed through an SCX cartridge, eluting with 2M NH_3 to yield **54** (910 mg, 3.1 mmol, 97 %). ^{1}H NMR (DMSO-d_6): δ 8.55 (s, 1H), 8.53 (s, 1H), 3.98 – 3.79 (m, 4H), 3.02 – 2.95 (m, 2H), 2.68 (d, *J* = 6.8 Hz, 2H), 2.59 – 2.52 (m, 2H, under solvent peak), 2.11 – 2.04 (m, 1H), 1.87 – 1.75 (m, 2H), 1.44 – 1.36 (m, 2H), 0.90 (d, *J* = 6.7 Hz, 6H)

***tert*-Butyl 2-chloro-5-(2-methoxyphenyl)-7*H*-pyrrolo[2,3-*d*]pyrimidine-7-carboxylate 67**

To **66** (600 mg, 1.80 mmol), (2-methoxyphenyl)boronic acid (274 mg, 1.80 mmol) and Pd(dppf)Cl_2 (73 mg, 0.09 mmol) in 1,4-Dioxane (10 mL) was added potassium phosphate (1.15 g, 5.4 mmol) in water (1 mL). The mixture was evacuated, flushed with argon and heated in a microwave (130 °C, 1 h). The mixture was partitioned between 20% aqueous NaCl and EtOAc, the organics evaporated and crude material purified by flash chromatography (0-50% EtOAc / hexane) to yield 2-chloro-5-(2-methoxyphenyl)-7*H*-pyrrolo[2,3-*d*]pyrimidine (300 mg, 1.16

mmol). MS (ESI): m/z 260.0 $[M + H]^+$. To this intermediate (180 mg, 0.69 mmol) in THF (10 mL) was added NaH (33 mg, 0.83 mmol) portion-wise, stirred for 10 min and Boc_2O (212 mg, 0.97 mmol) added. The mixture was stirred overnight, quenched by drop-wise addition of water, partitioned between water / DCM and the organics evaporated and purified by flash chromatography (0 – 40% EtOAc/ hexane) to yield **67** (230 mg, 0.64 mmol, 92%) which was used in the subsequent step without further purification. MS (ESI): m/z 360.1, 362.1 $[M + H]^+$.

***tert*-Butyl 4-((4-methoxy-5-(morpholine-4-carbonyl)pyrimidin-2-yl)amino)piperidine-1-carboxylate 69**

To a solution of *tert*-butyl 4-aminopiperidine-1-carboxylate (6.72 g, 33.53 mmol) and DIPEA (12.75 mL, 73 mmol) in 1,4-dioxane (140 mL) was added (2-Chloro-4-methoxypyrimidin-5-yl)(morpholino)methanone (6.27 g, 24.33 mmol). The mixture was heated to reflux for 17 h, solvent was evaporated and partitioned between sat'd aqueous NH4Cl / DCM and the organics dried over Na_2SO_4 and evaporated to give crude product which was purified by flash chromatography (0 – 30% 2-propanol / cyclohexane) to give **69** (6.83 g, 16.20 mmol, 67 %). ^1H NMR ($CDCl_3$) δ 8.23 – 8.13 (m, 1H), 5.29 – 4.95 (m, 1H), 4.16 – 3.99 (m, 4H), 3.96 (s, 3H), 3.86 – 3.61 (m, 6H), 3.43 – 3.33 (m, 1H), 3.03 – 2.93 (m, 2H), 2.10 – 2.03 (m, 2H), 1.51 (s, 9H), 1.48 – 1.41 (m, 2H). MS (ESI): m/z 422.3 $[M + H]^+$.

***tert*-Butyl 4-((4-methoxy-5-(morpholine-4-carbonothioyl)pyrimidin-2-yl)amino)piperidine-1-carboxylate 70**

To a solution **69** (6.8 g, 16.13 mmol) in THF (100 mL) was added Lawesson's reagent (6.53 g, 16.13 mmol) and the mixture stirred at 50 °C for 1.5 h. Sat'd aqueous $NaHCO_3$ (200 mL) was added and the aqueous layer extracted with EtOAc (250 mL). The organic phase was washed with brine (200 mL), dried over Na_2SO_4 and solvent evaporated to give crude product which was purified by flash chromatography (0 – 30% 2-propanol / cyclohexane) to give **70** (6.1 g, 13.94 mmol, 86%). ^{1}H NMR ($CDCl_3$) δ 8.32 (s, 1H), 5.28 – 5.03 (m, 1H), 4.56 – 4.48 (m, 1H), 4.40 – 4.30 (m, 1H), 4.13 – 4.04 (m, 2H), 4.03 – 3.99 (m, 1H), 3.96 (s, 3H), 3.92 – 3.87 (m, 2H), 3.82 – 3.74 (m, 1H), 3.70 – 3.58 (m, 2H), 3.56 – 3.48 (m, 1H), 3.05 – 2.92 (m, 2H), 2.08 – 2.02 (m, 2H), 1.51 (s, 9H), 1.48 – 1.39 (m, 2H). MS (ESI): m/z 438.2 $[M + H]^+$.

***tert*-Butyl 4-((3-morpholino-1*H*-pyrazolo[3,4-*d*]pyrimidin-6-yl)amino)piperidine-1-carboxylate 71**

To a stirred suspension of **70** (6.0 g, 13.71 mmol) in dioxane (100 mL) was added hydrazine monohydrate (13.3 mL, 274 mmol) and the mixture stirred at 100 ºC for 4 h. The solvent was evaporated and the crude material triturated with ether to give **71** (5.22 g, 12.94 mmol, 94 %). ^{1}H NMR (DMSO-d_6) δ 12.03 (br s, 1H), 8.83 (s, 1H), 7.27 (br s, 1H), 3.92 (d, J = 12.4 Hz, 3H), 3.76 – 3.72 (m, 4H), 3.32 – 3.28 (m, 4H), 3.00 – 2.70 (m, 2H), 1.92 – 1.79 (m, 2H), 1.41 (s, 9H), 1.39 – 1.29 (m, 2H). MS (ESI) m/z 404.2 $[M + H]^+$.

3-Morpholino-*N*-(piperidin-4-yl)-1*H*-pyrazolo[3,4-*d*]pyrimidin-6-amine72

To a suspension of **71** (1.0 g, 2.48 mmol) in EtOH (10 mL) was added HCl (3M in MeOH, 21 mL, 62.0 mmol) and the mixture stirred at 60 ºC for 2 h. Volatiles were evaporated to afford **72**

(HCl salt, 0.9 g, 2.65 mmol, quant.) which was used without purification. ^{1}H NMR (DMSO-d_6) δ 12.85 (br s, 1H), 9.15 (br s, 1H), 9.07 – 8.93 (m, 1H), 8.91 – 8.68 (m, 1H), 8.59 – 8.12 (m, 1H), 4.16 – 3.98 (m, 1H), 3.81 – 3.69 (m, 4H), 3.39 – 3.34 (m, 4H), 3.30 (d, *J* = 13.1 Hz, 2H), 3.10 – 2.96 (m, 2H), 2.14 – 2.02 (m, 2H), 1.82 – 1.70 (m, 2H). MS (ESI) m/z 304.2 $[M + H]^+$.

NMR and LCMS for Selected Compounds

Compound 8

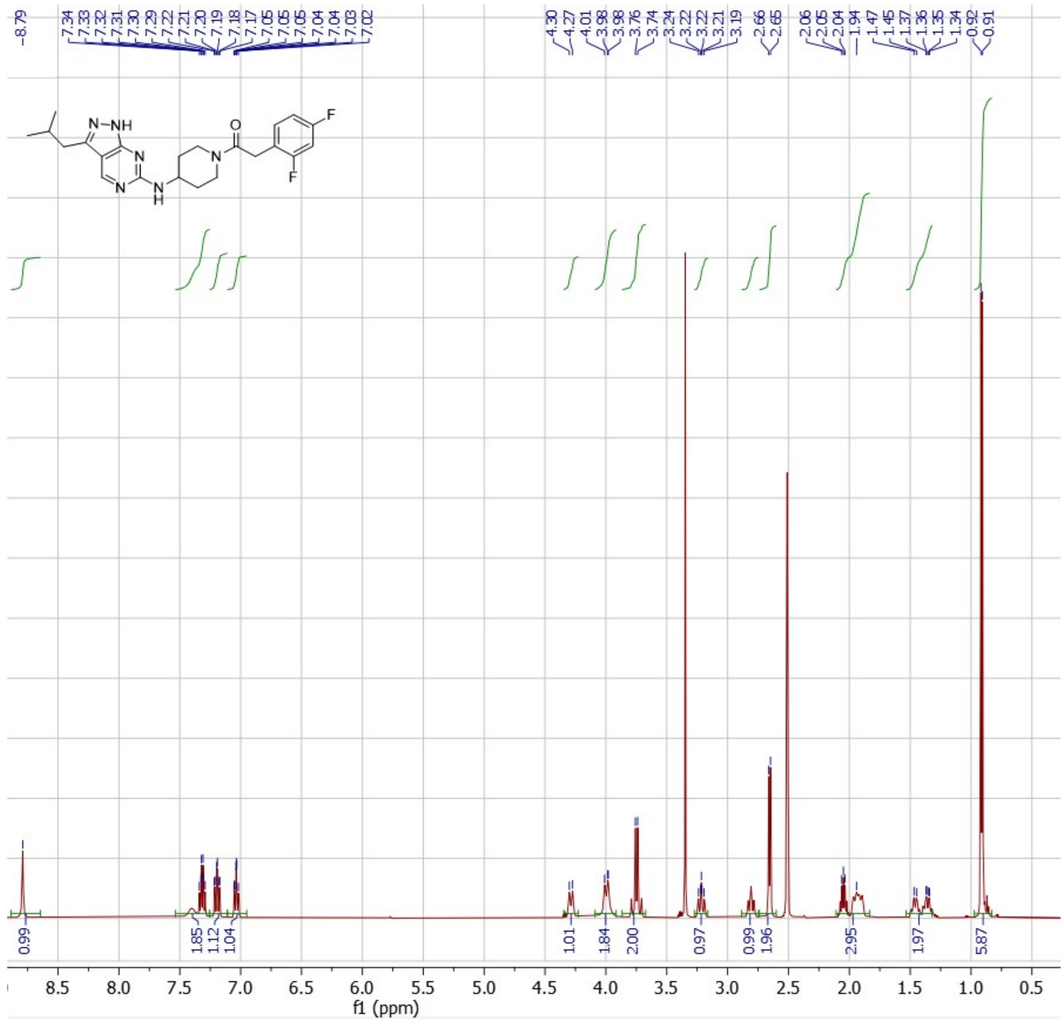

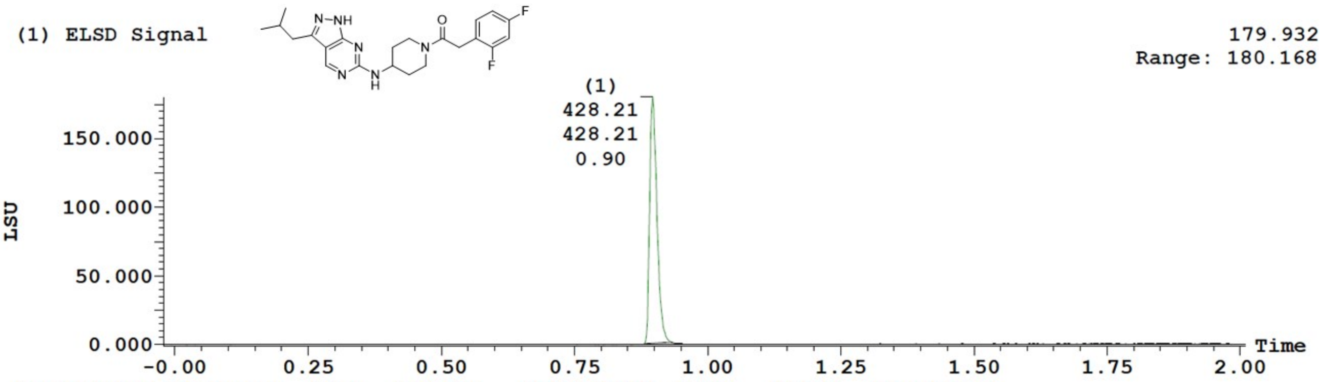

Peak Number	Compound	Time	AreaAbs	Area %Total	Width	Height	Mass Found
1	Found	0.90	3e+003	100.00	0	2e+005	428.21, 428.21

Compound 13

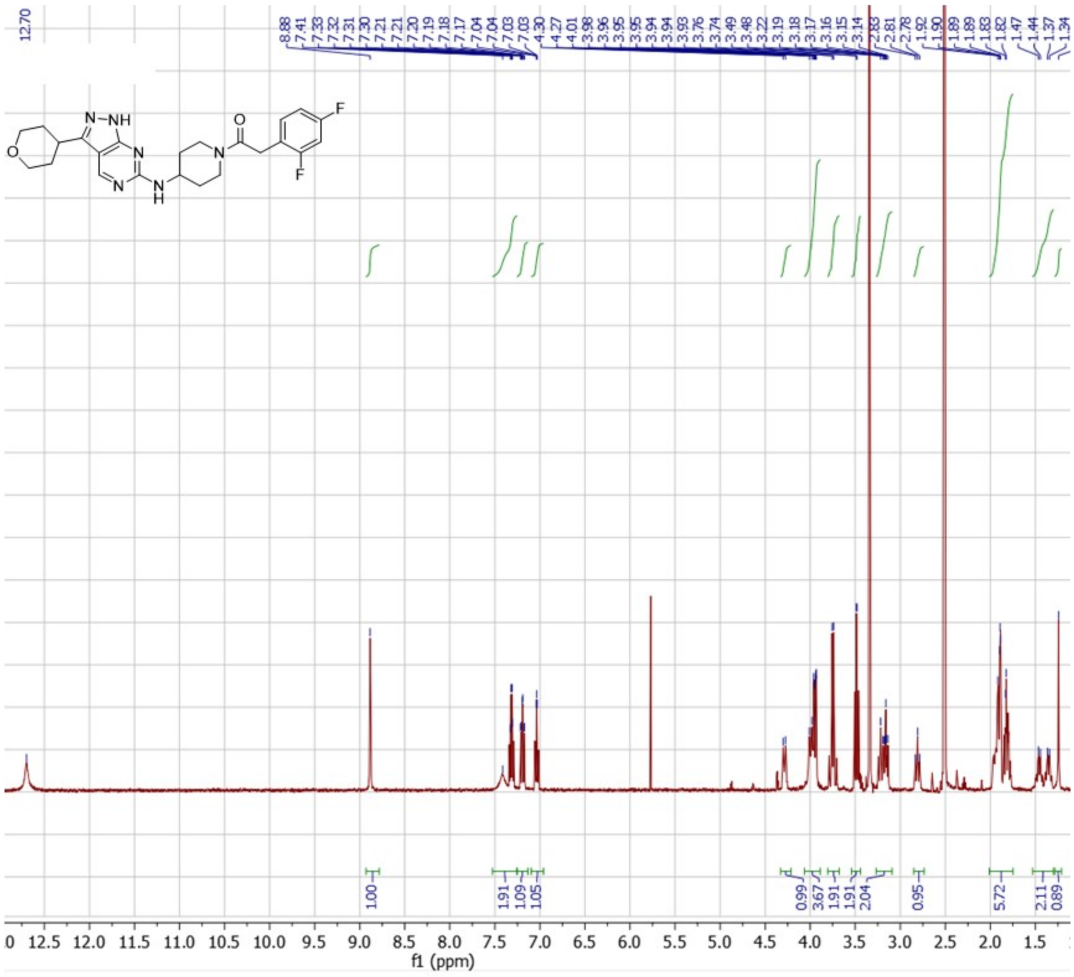

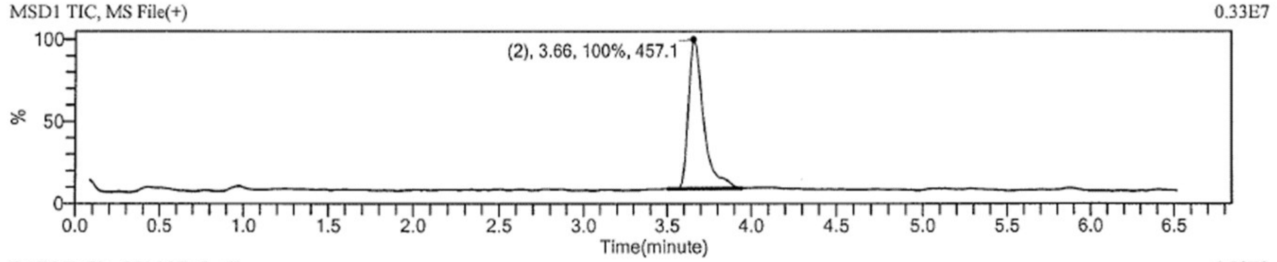
MSD1 TIC, MS File(+)
0.33E7
(2), 3.66, 100%, 457.1
%
Time(minute)

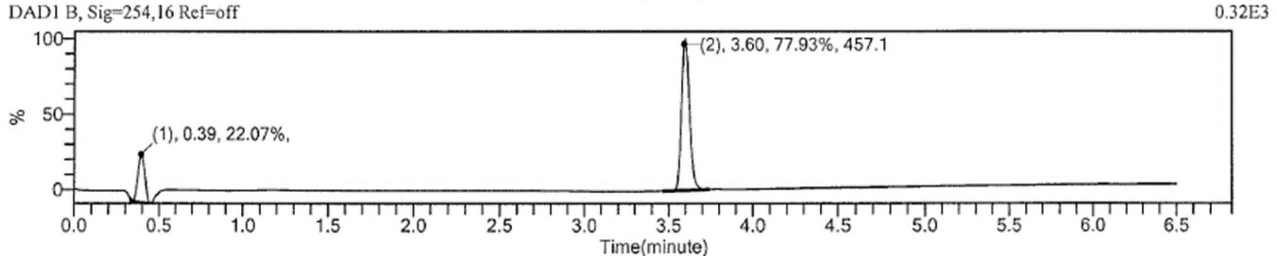
DAD1 B, Sig=254,16 Ref=off
0.32E3
(2), 3.60, 77.93%, 457.1
(1), 0.39, 22.07%,
%
Time(minute)

Compound 15

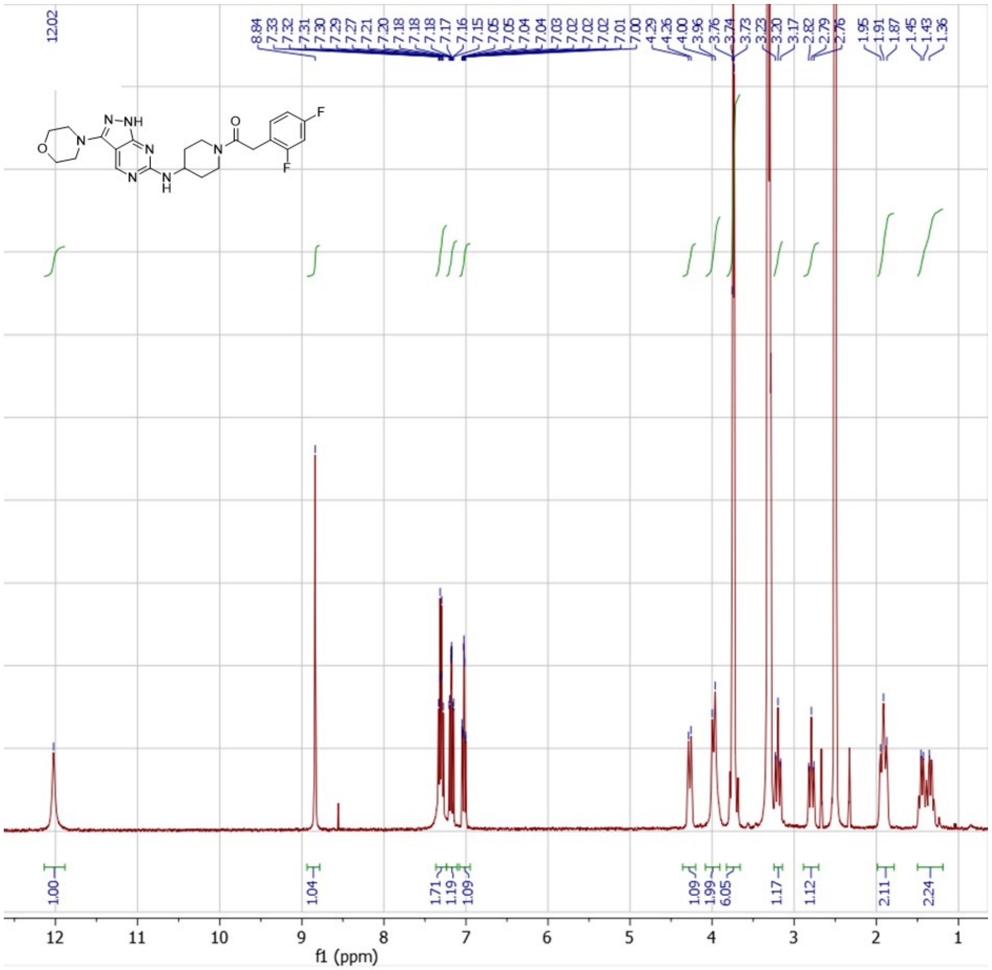
12.02
8.84
4.29
4.26
4.00
3.96
3.76
3.74
3.73
3.23
3.20
3.17
2.82
2.79
2.76
1.95
1.91
1.87
1.45
1.43
1.36
1.00
1.04
1.71
1.19
1.09
1.09
1.99
6.05
1.17
1.12
2.11
2.24
f1 (ppm)

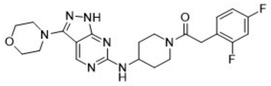

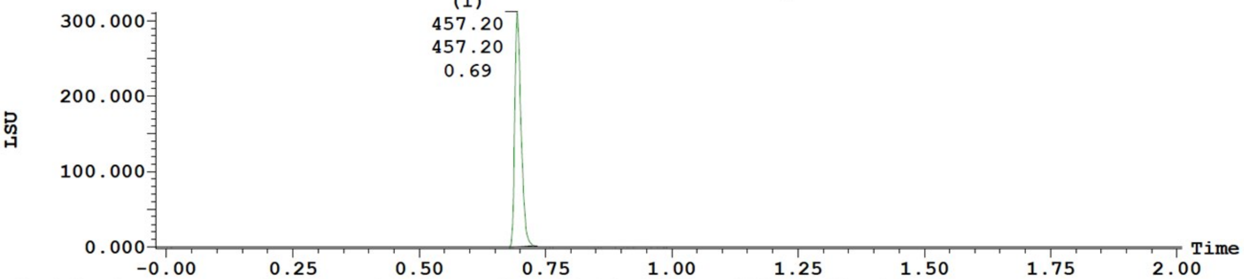

Peak Number	Compound	Time	AreaAbs	Area %Total	Width	Height	Mass Found
1	Found	0.69	4e+003	100.00	0	3e+005	457.20, 457.20

Compound 20

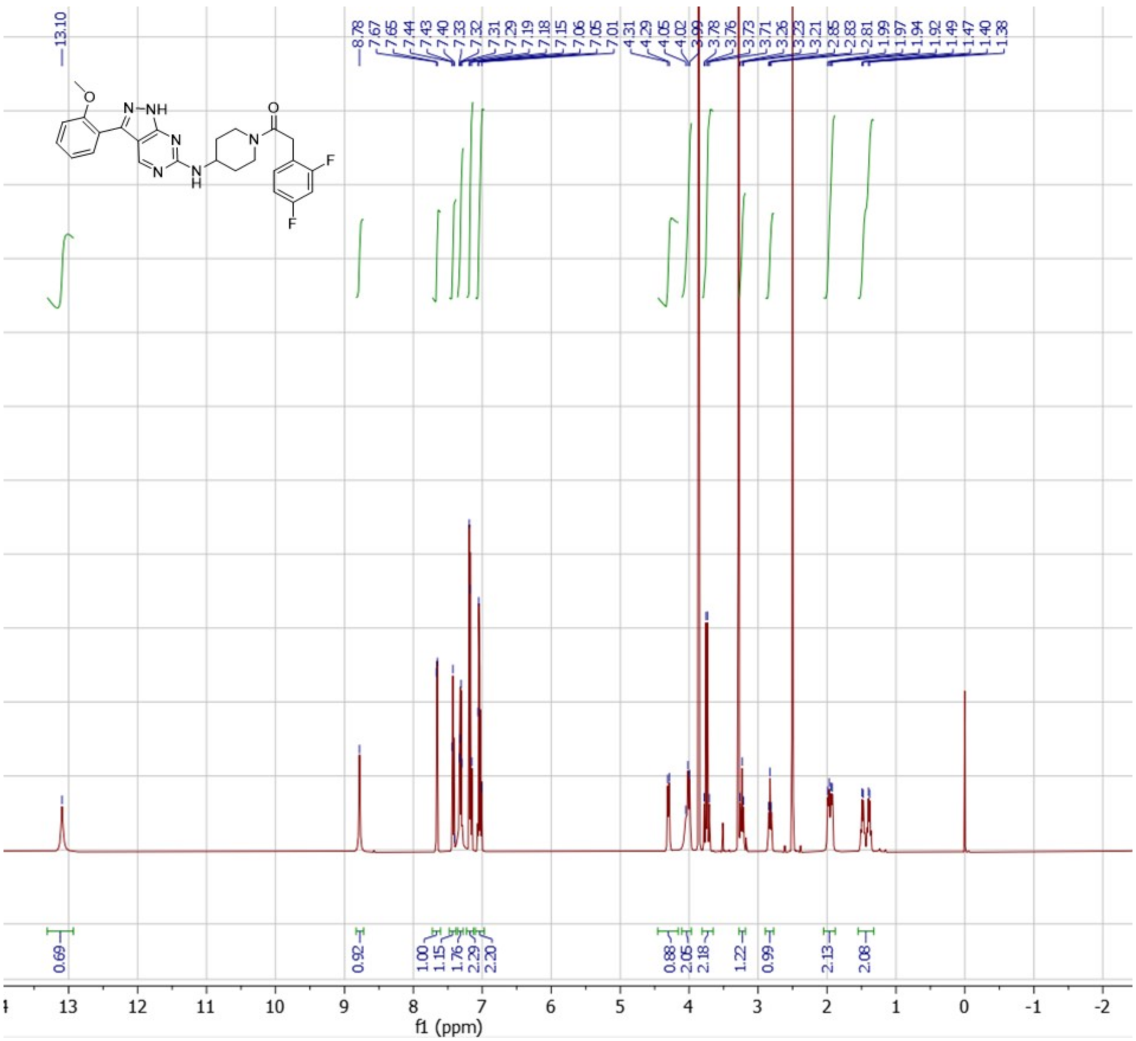

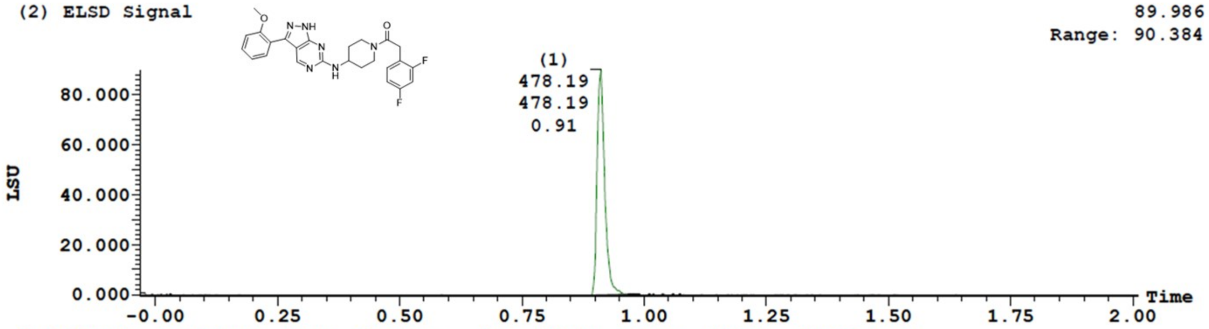

Peak Number	Compound	Time	AreaAbs	Area %Total	Width	Height	Mass Found
1	Found	0.91	2e+003	100.00	0	9e+004	478.19, 478.19

Compound 37

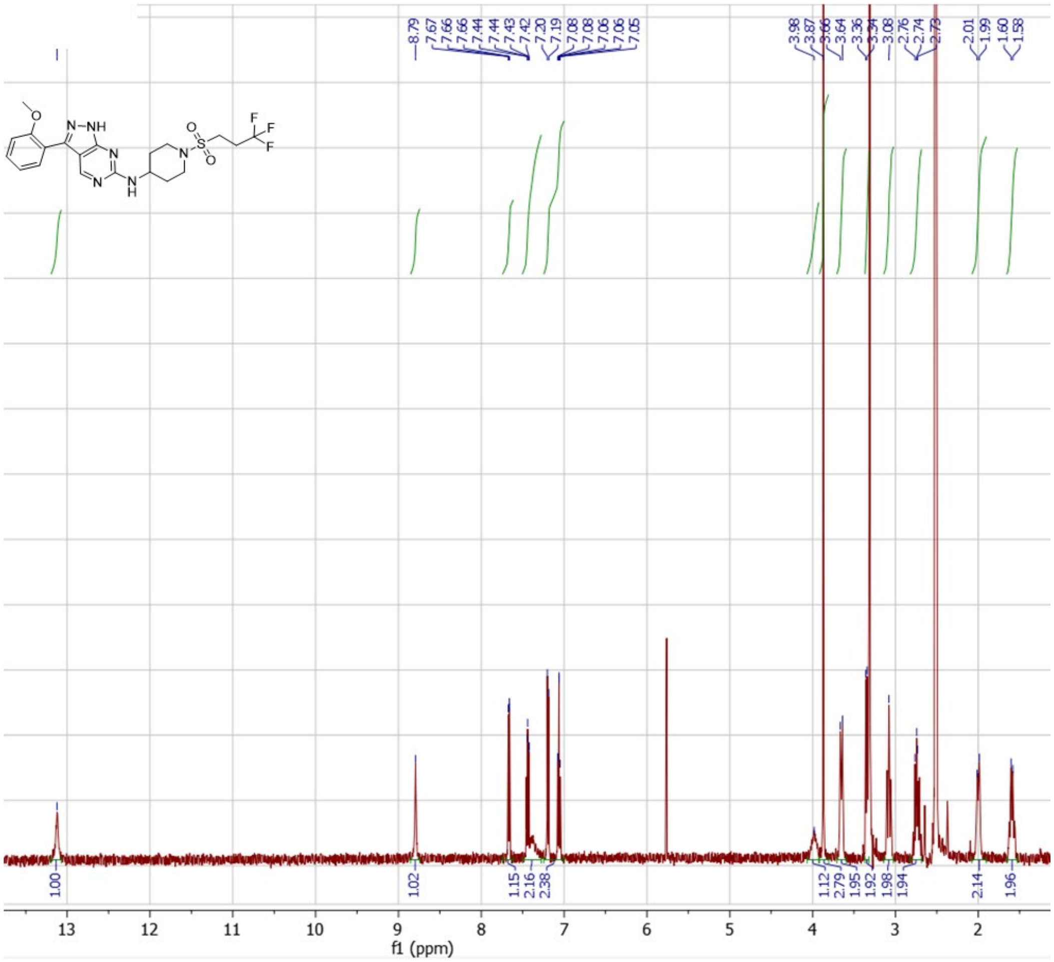
8.79
7.67
7.66
7.66
7.44
7.43
7.42
7.20
7.19
7.08
7.08
7.06
7.06
7.05
3.98
3.87
3.66
3.64
3.36
3.34
3.08
2.76
2.74
2.73
2.01
1.99
1.60
1.58
1.00
1.02
1.15
2.16
2.38
1.12
2.79
1.95
1.92
1.98
1.94
2.14
1.96
13
12
11
10
9
8
7
6
5
4
3
2
f1 (ppm)

Compound 40

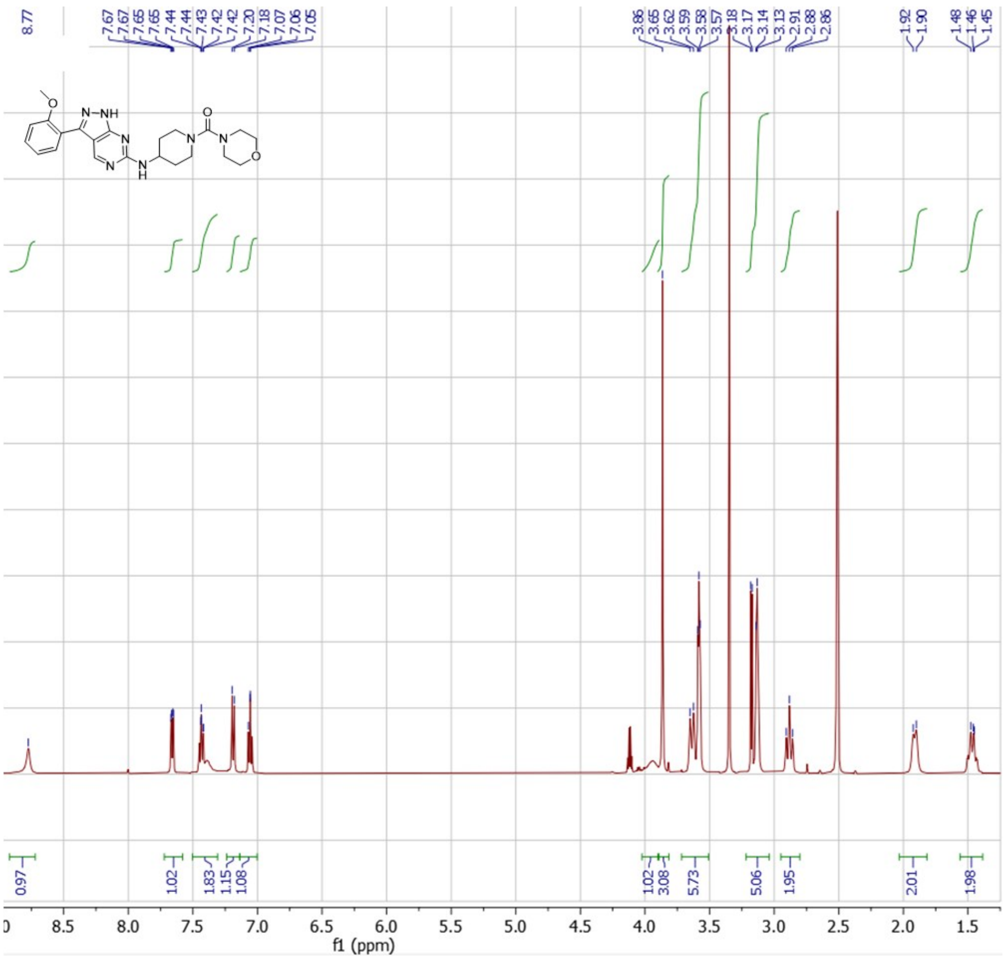

8.77
7.67
7.67
7.65
7.65
7.44
7.44
7.43
7.42
7.42
7.20
7.18
7.07
7.06
7.05
3.86
3.65
3.62
3.59
3.58
3.57
3.18
3.17
3.14
3.13
2.91
2.88
2.86
1.92
1.90
1.48
1.46
1.45
0.97
1.02
1.83
1.15
1.08
1.02
3.08
5.73
5.06
1.95
2.01
1.98
8.5
8.0
7.5
7.0
6.5
6.0
5.5
5.0
4.5
4.0
3.5
3.0
2.5
2.0
1.5
f1 (ppm)

Compound 42

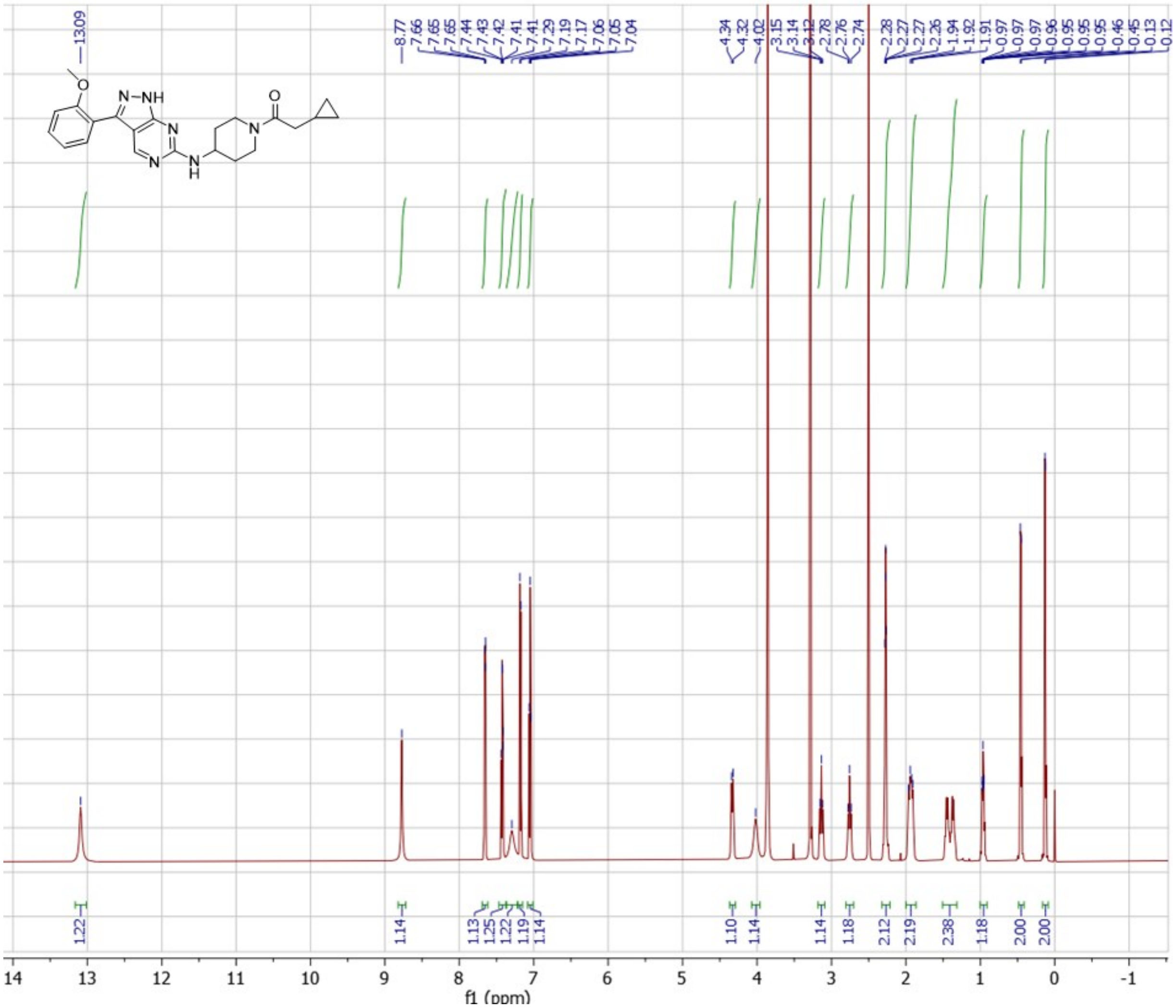

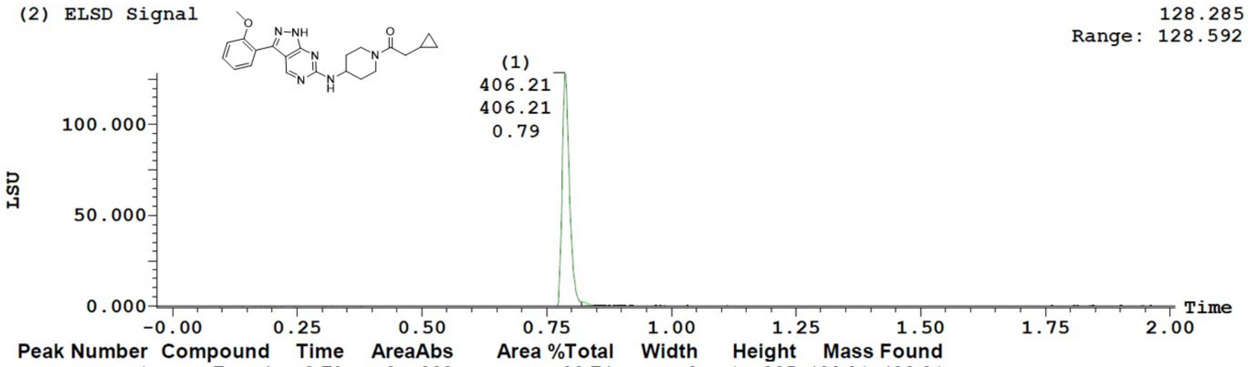

Peak Number	Compound	Time	AreaAbs	Area %Total	Width	Height	Mass Found
1	Found	0.79	2e+003	98.74	0	1e+005	406.21, 406.21
2	Found	0.82	3e+001	1.26	0	2e+003	406.21, 406.21

Compound 43

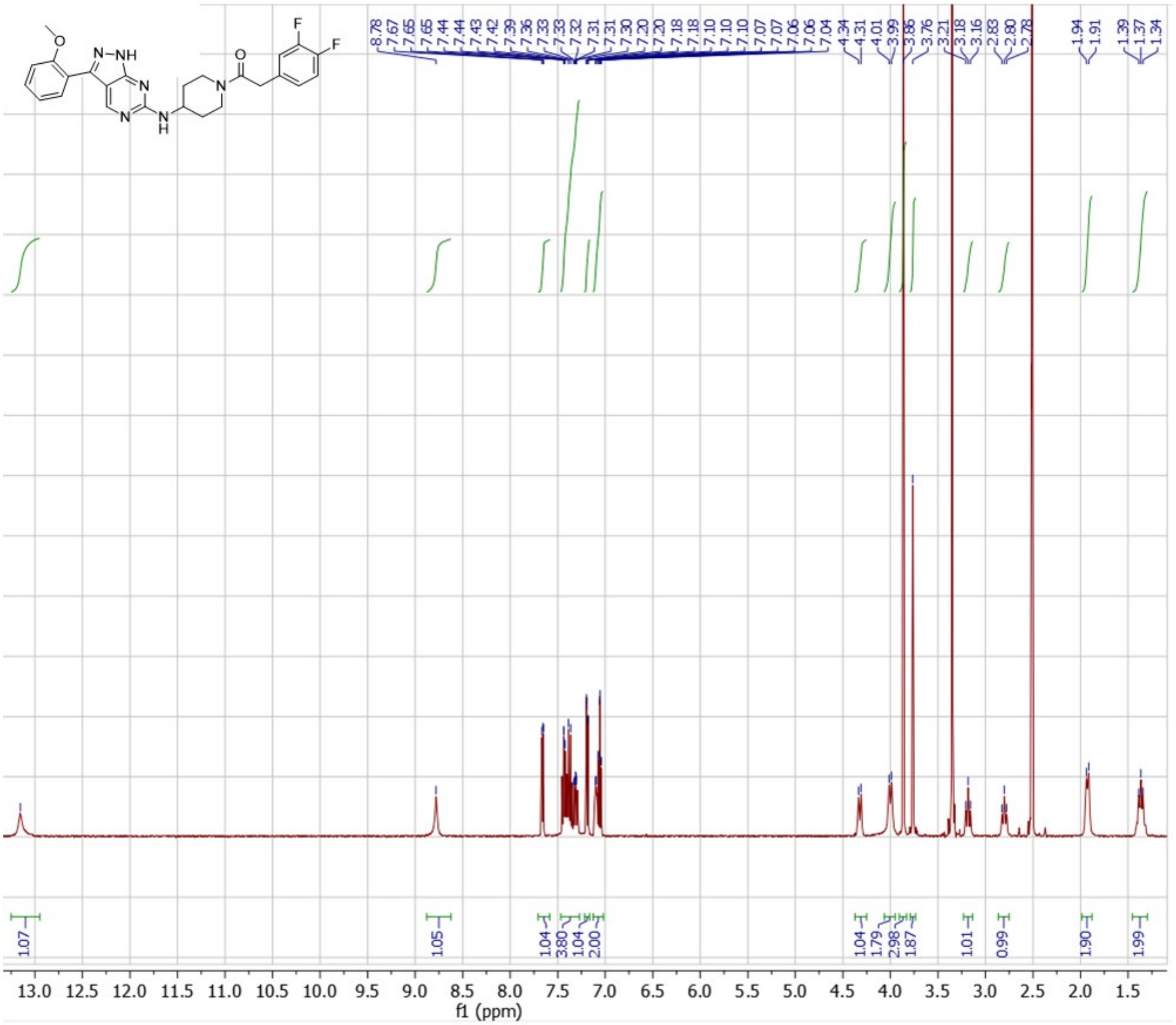

Compound 48

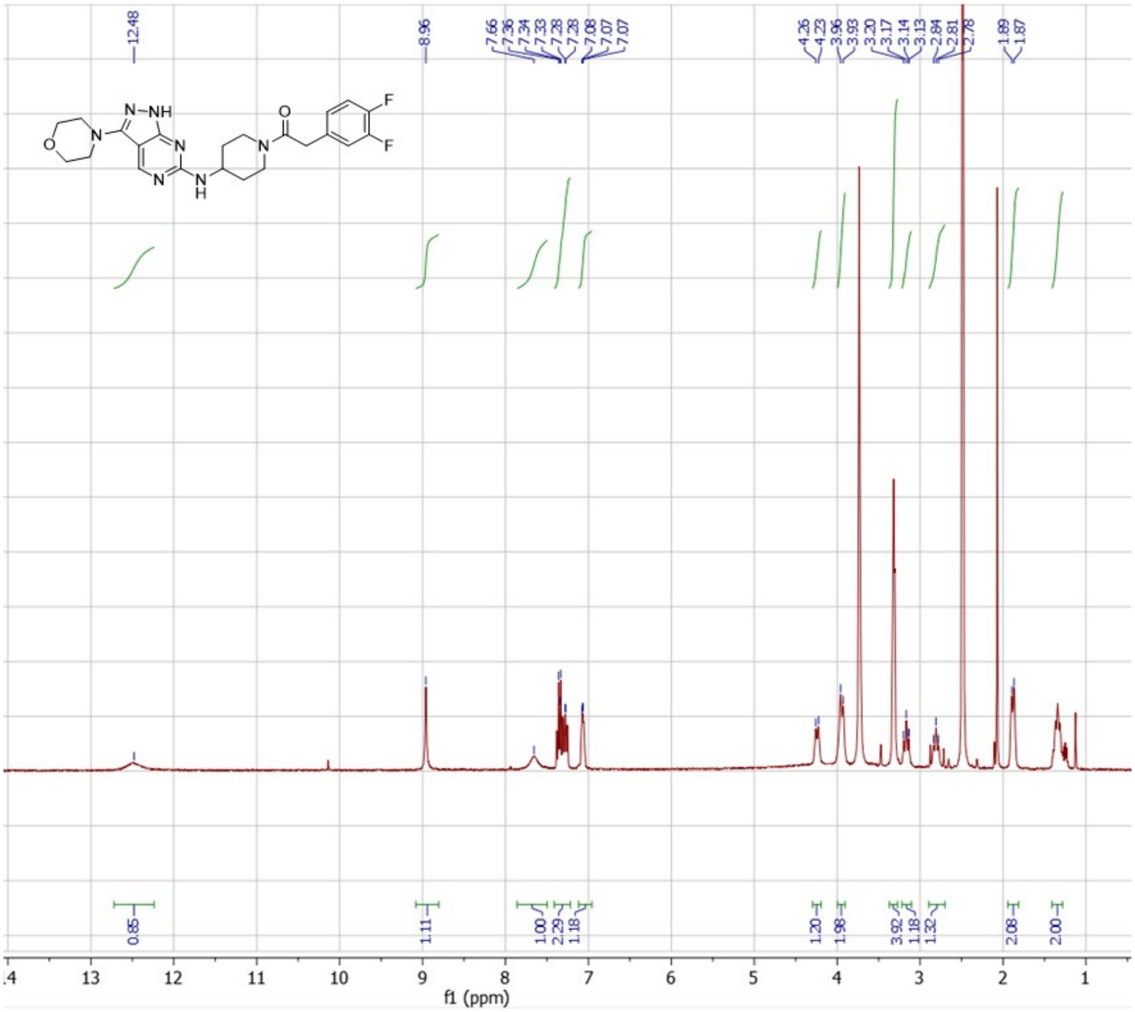

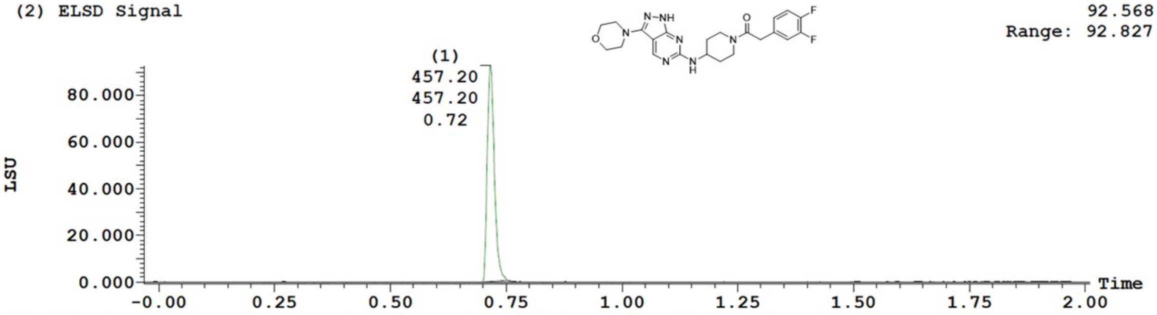

Peak Number	Compound	Time	AreaAbs	Area %Total	Width	Height	Mass Found
1	Found	0.72	2e+003	100.00	0	9e+004	457.20, 457.20

Compound 50

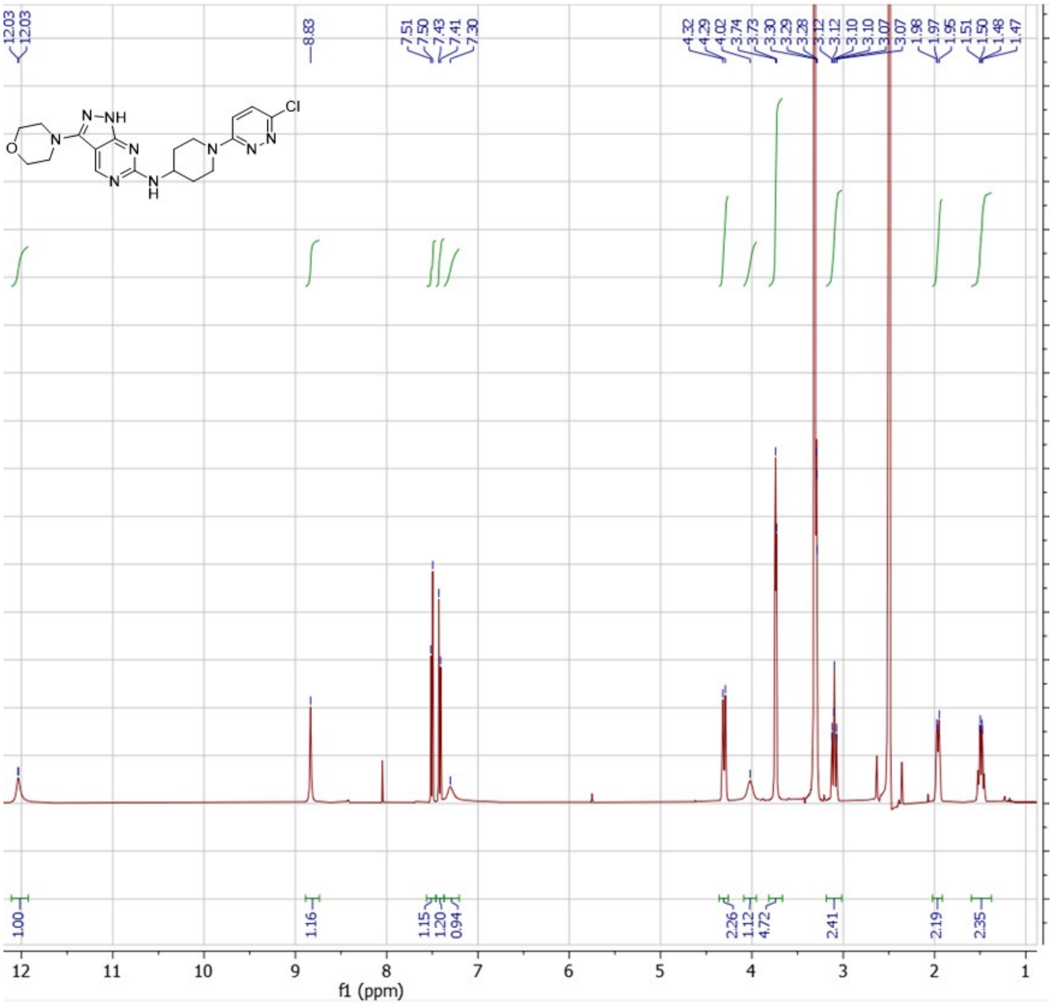

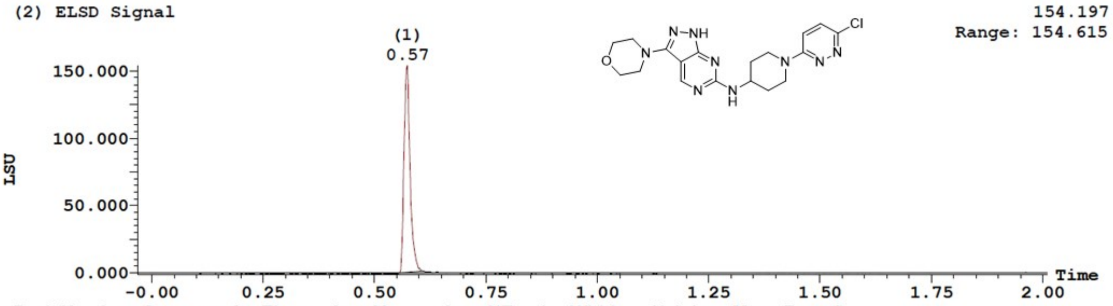

Peak Number	Compound	Time	AreaAbs	Area %Total	Width	Height	Mass Found
1		0.57	2e+003	100.00	0	2e+005	

Peak ID	Compound	Time	Mass Found
1		0.58	

SAMPLE: 1:4,14 Combine (76:81-(46:49+111:113))

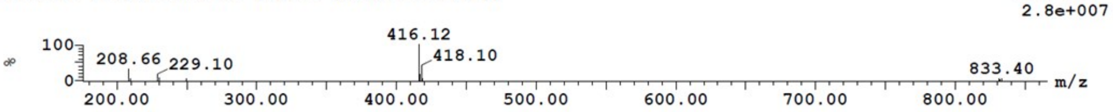

Assay Protocols

Intramacrophage Leishmania donovani Assay

This assay was conducted as previously described [9]

CAD Aqueous Solubility [11]

5µl of 10mM DMSO stock solution is diluted to 100 µl with pH7.4 phosphate buffered saline, equilibrated for 1 hour at room temperature and filtered through Millipore MultiscreenHTS-PCF filter plates (MSSL BPC). The filtrate is quantified by suitably calibrated Charged Aerosol Detector

Stability in Microsomes [10]

Test compound (0.5 µM) was incubated with female CD1 mouse (Xenotech) liver microsomes and their action started with addition of excess NADPH (8 mg/mL 50 mM potassium phosphate buffer, pH 7.4). Immediately, at time zero, then at 3, 6, 9, 15, and 30 min, an aliquot (50 µL) of the incubation mixture was removed and mixed with acetonitrile (100 µL) to stop the reaction. Internal standard was added to all samples, the samples centrifuged to sediment precipitated protein, and the plates then sealed prior to UPLC-MS/MS analysis using a Xevo TQs micro (Waters Corporation, USA). XLfit (IDBS, UK) was used to calculate the exponential decay and consequently the rate constant (k) from the ratio of the peak area of test compound to internal standard at each time point. The rate of intrinsic clearance (Cl_i) of each test

compound was then calculated using the equation Cl_i (mL/min/g liver) = k × V × microsomal protein yield, where V (mL/mg protein) is the incubation volume/mg protein added and

microsomal protein yield is taken as 52.5 mg protein/g liver. Verapamil (0.5 μM) was used as a positive control to confirm acceptable assay performance.

***In Vivo* Efficacy Studies** [10]

Groups of female BALB/c mice (5 per group) were inoculated intravenously with approximately 2 × 107 *L. donovani* amastigotes (LV9; WHO designation: MHOM/ET/67/HU3) harvested from the spleen of an infected hamster. From day 7 post infection, groups of mice were treated with either drug vehicle only (orally), with control drugs Miltefosine 30 mg/kg orally and Pentostam 15 mg/kg subcutaneously (only in compound 48 efficacy experiment) or with test compound at the appropriate dose. Miltefosine and Pentostam were dosed once daily for 5 days and test compounds were dosed twice daily for 5 days. Drug dosing solutions were prepared fresh each day and the vehicle was deionized water for Miltefosine and Pentostam, or 0.5% HPMC, 0.4% Tween 80, and 0.5% benzyl alcohol for test compounds. On day 14 all animals were humanely euthanized, liver smears made, and parasite burdens determined by counting the number of amastigotes/500 liver cells. Parasite burden is expressed in Leishman−Donovan units (LDU): the number of amastigotes per 500 nucleated cells multiplied by the organ weight in milligrams.
